# An integrated platform for genome engineering and gene expression perturbation in *Plasmodium falciparum*

**DOI:** 10.1038/s41598-020-77644-4

**Published:** 2021-01-11

**Authors:** Armiyaw S. Nasamu, Alejandra Falla, Charisse Flerida A. Pasaje, Bridget A. Wall, Jeffrey C. Wagner, Suresh M. Ganesan, Stephen J. Goldfless, Jacquin C. Niles

**Affiliations:** Department of Biological Engineering, Massachusetts Institute of Technology, 77 Massachusetts Avenue, Room 56-341B, Cambridge, MA 02139 USA

**Keywords:** Parasite genetics, Functional genomics, Microbial genetics

## Abstract

Establishing robust genome engineering methods in the malarial parasite, *Plasmodium falciparum*, has the potential to substantially improve the efficiency with which we gain understanding of this pathogen’s biology to propel treatment and elimination efforts. Methods for manipulating gene expression and engineering the *P. falciparum *genome have been validated. However, a significant barrier to fully leveraging these advances is the difficulty associated with assembling the extremely high AT content DNA constructs required for modifying the *P. falciparum *genome. These are frequently unstable in commonly-used circular plasmids. We address this bottleneck by devising a DNA assembly framework leveraging the improved reliability with which large AT-rich regions can be efficiently manipulated in linear plasmids. This framework integrates several key functional genetics outcomes via CRISPR/Cas9 and other methods from a common, validated framework. Overall, this molecular toolkit enables *P. falciparum *genetics broadly and facilitates deeper interrogation of parasite genes involved in diverse biological processes.

## Introduction

Malaria is an infectious disease caused by *Plasmodium species* parasites and continues to be a leading cause of morbidity and mortality worldwide. In 2019, there were over 229 million malaria cases and 409,000 deaths due mostly to *Plasmodium falciparum* infections ^1^. Basic molecular understanding of these parasites is still limited, and this impedes efforts to discover new therapeutic approaches to preventing, treating and eliminating malaria. The genomes of several *Plasmodium* species have now been sequenced, and nearly half of the identified genes are of uncharacterized function ^2^. Therefore, functional genetics studies will be vital for systematically defining the roles of the various parasite genes toward improving our overall understanding of *Plasmodium* parasite biology. A key requirement for maximizing the effectiveness of this approach is the ability to selectively perturb gene expression to study biological function in detail.

Several recently-completed genome-scale studies have provided novel and substantial insights towards categorizing genes that are likely essential versus dispensable to survival of *Plasmodium spp* ^3,4^, and the related Apicomplexan parasite, *Toxoplasma gondii* ^5^. The first comprehensive dataset came from a genome-wide CRISPR/Cas9 deletion screen in the genetically-tractable *T. gondii*. Homologous recombination-mediated knockout screens in the model rodent malaria parasite *Plasmodium berghei* ^3^ and piggyBac saturation insertional mutagenesis screens in *P. falciparum* ^4^ have also provided unprecedented insight into the complement of likely essential genes during asexual blood stage development in these comparably less genetically-tractable organisms. The above screening approaches facilitate identification of likely essential genes through the inability to recover surviving parasites mutated or deleted in a given locus. As such, stable cell lines with disrupted essential gene loci are usually not recovered, and this precludes direct follow-up to elucidate function. Therefore, validating the findings of these high throughput screens, and achieving detailed understanding of biological function requires implementing scalable approaches that harness methods for conditionally perturbing the expression of a rapidly expanding number of identified essential genes. Several factors impede this process. First, the most robust and reliable conditional gene expression systems used currently in *Plasmodium* parasites require genetically encoding information at native chromosomal loci using homology-based donor vectors ^6–10^. Second, a genomic AT content exceeding 80% ^2^ complicates efficient, scalable assembly of the DNA vectors needed to achieve these modifications. Third, relatively inefficient recombination events have typically been used to modify target loci in a protracted process ^11^. Combined, these factors complicate efforts to genetically modify many gene loci in parallel. Successful genome editing in *P. falciparum* using zinc finger nucleases (ZFNs) ^12^ and CRISPR/Cas9 ^13,14^ provide attractive solutions for improving the ease with which targeted gene locus modification can be achieved. However, scalable and efficient application of these approaches will require facile and reliable construction of AT-rich, homology-based vectors suitable for delivering the desired final functional modifications.

Here we demonstrate a highly adaptable framework that flexibly provides reliable assembly of *P. falciparum* vectors to support stable episomal maintenance of bacterial artificial chromosomes (BACs), genome modification by single- and double-crossover integration and *Bxb1*-mediated integration ^15^ and gene-specific editing using CRISPR/Cas9 strategies. To complement CRISPR/Cas9 genome editing experiments, we have generated a parasite cell line stably expressing the Cas9 editing machinery, which serves as a convenient alternative to co-delivery of Cas9 during transfection. We include within this framework the abilities to flexibly epitope tag, conditionally regulate, knockout and/or complement/overexpress target genes. We show that assembly of large, AT-rich regions typically encountered when creating *P. falciparum* expression and homology-directed repair vectors is readily achieved. The vectors produced using this approach successfully yield transgenic parasites in which genetic elements are either episomally maintained or chromosomally integrated as pre-specified, and that pre-installed regulatory components function as expected. Altogether, this harmonized functional genetics toolbox represents a well-validated and standardized resource that will be useful for meeting the growing and diverse needs of *P. falciparum* functional genetics.

## Results and discussion

### Rationale motivating vector assembly framework

Several challenges associated with vector assembly limit the ease and scale of target-specific functional genetics studies in *P. falciparum* ^16^. We defined key limitations to overcome and useful enabling functionalities to capture in our designs. We identified the need for a robust cloning chassis that supports easy manipulation and stable maintenance of AT-rich *P. falciparum* genomic DNA of broad size ranges. This permits unconstrained user selection of DNA fragment sizes best suited to the downstream application. When AT-rich sequences are present in the typical circular plasmids used to generate transgenic *P. falciparum*, they are often and unpredictably deleted and/or induce rearrangements during propagation in *E. coli* ^17^. To overcome this, we selected the linear pJAZZ plasmid vector (Lucigen) as the chassis for all routine DNA assembly operations, as it has been used to successfully manipulate large AT-rich genomic fragments, including those derived from the related model rodent malaria parasite, *P. berghei* ^17,18^. We reasoned that this context would allow rapid and modular DNA assembly to be completed with high fidelity and improve overall vector construction efficiency.

While linear plasmids can facilitate accelerated DNA part assembly, only circular plasmids are stably replicated episomally in *P. falciparum* ^19^. Thus, to retain this option, a strategy for efficiently converting linear plasmids into circular forms while avoiding undesirable rearrangements is beneficial. Supercoiling in circular plasmids induces single-stranded regions within AT-rich sequences making these susceptible to nicking and deleterious rearrangements that reduce torsional stress ^20^. Therefore, rather than converting linear vectors into plasmids, we reasoned that rescuing linear vectors containing AT-rich sequences into larger BACs where supercoiling-induced torsional stress and plasmid instability are inherently lower would be highly effective. In other contexts, uncomplicated by highly AT-rich sequences, this approach has been used successfully ^21^. We anticipate that establishing similar approaches for *P. falciparum* will facilitate using standardized procedures to streamline and scale up production of vectors needed to pursue a range of functional genetics studies.

### Large DNA fragments containing multiple, AT-rich *P. falciparum* gene expression regulatory regions are readily ported to linear vectors

**Figure 1:**
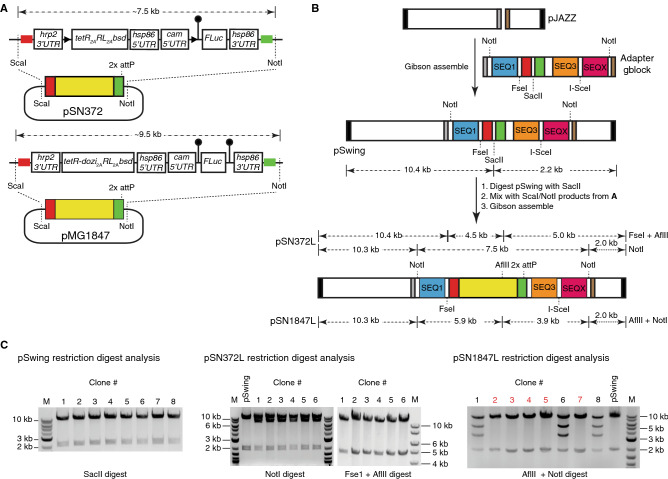
Proof-of-concept to establish successful transfer of large DNA fragments containing interspersed regions of AT-rich regulatory elements to a linear vector framework. (**A**). The schematic shows 7.5 kb and 9.5 kb fragments to be released from extant circular vectors pSN372 and pMG1847, respectively, for assembly into linear vectors. These fragments contain TetR- or TetR-DOZI-based translation regulation modules and a transcriptional unit in which expression of a FLuc reporter CDS is translationally controlled by TetR aptamers (shown as lollipops) located in either the 5′-*UTR* only or both 5′- and 3′-*UTR*s. (**B**) Strategy used to transfer the respective pSN372- and pMG1847-derived fragments into linear plasmids. The original pJAZZ-OC vector (Lucigen) was modified with a multi-cloning site gene block to create pSwing. To facilitate Gibson assembly, pSwing can be digested with restriction enzymes to expose regions homologous to cut pSN372- and pMG1847-derived fragments (red and green). (**C**) Restriction digestion analysis confirming proper topological assembly of pSwing, pSN372L and pSN1847L. For pSN1847L, several plasmids that do not contain the expected insert, and likely corresponding to pSwing, are indicated in red font.

We examined the feasibility of rapidly transferring large, pre-existing DNA fragments into this linear plasmid format. This option permits direct transfer of existing parts to this new framework, while preserving access to existing user-preferred features. To demonstrate this, we transferred two configurations of our validated TetR-aptamer regulatory system that we also intended to hardwire into all future linear vector designs (Fig. 1A). In the first case, an ~ 7.5 kb fragment consisting of two head-to-head *P. falciparum* cassettes was transferred. One cassette encodes TetR, *Renilla* luciferase (RL) and Blasticidin S deaminase (*bsd*) as a multicistronic message using the viral 2A peptide ^12,16^. The other cassette encodes firefly luciferase (FLuc) regulated by a single TetR aptamer in its 5′*UTR* ^7^. In the second case, an ~ 9.5 kb fragment similar to that described above was migrated, except that the TetR component in the first cassette is replaced by a TetR–DOZI fusion, and an array of ten tandem TetR aptamers is included just upstream of the 3′*UTR* in the FLuc cassette ^8^.

**Figure 2:**
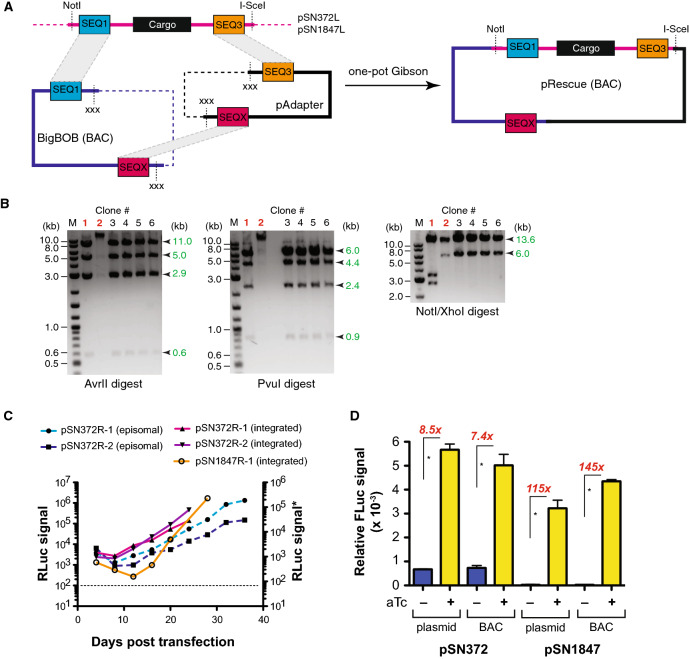
Establishing successful rescue of *P. falciparum* regulatory modules assembled in linear vectors to BACs. (**A**) Schematic of the linear vector rescue process, wherein the linear vector is digested with NotI/I-Sce1 to expose the SEQ1 and SEQX regions at the ends. The recipient BAC, BigBOB, and pAdapter plasmid are pre-configured such that the SEQ1/SEQ3 and SEQ3/SEQX regions can be exposed upon restriction enzyme digestion with PacI and XbaI/XhoI, respectively. The appropriate fragments from the linear plasmid, BigBOB BAC and pAdapter restriction enzyme digestions are mixed and assembled in a single pot, 3-piece Gibson reaction. (**B**) Restriction enzyme digestion mapping, illustrated for pSN372R, is used to identify BACs with the expected topology. Examples of BACs isolated from transformation of the same Gibson assembly reaction that were correctly (black font) versus incorrectly (red font) assembled are shown. (**C**) Renilla luciferase measurements were monitored to successful transfection of *P. falciparum* using rescued BACs. *Note: Renilla luciferase measurements shown in solid and dashed lines were made with the Promega Dual-Luciferase and Renilla luciferase Assay Systems, respectively. Parasitemia values generally reached ~ 0.5–1% by ~ 20–24 days post-transfection (**D**) Comparison of conditional regulation of normalized FLuc expression from plasmid and BAC contexts for pSN372 and pSN1847. Data are the mean of *n* = 3 ± standard deviation for each condition. * *p* ≤ 0.05 by Student’s t-test.

**Figure 3:**
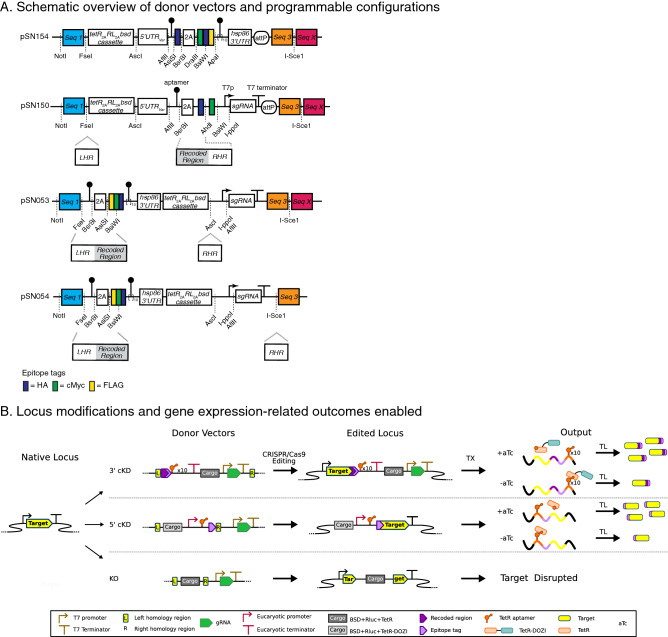
Overview of some key strategies used and capabilities accessible using the current integrative framework. (**A**) Schematic illustrations of linear vectors pSN150, pSN154, pSN053 and pSN054 summarizing some key functional features and restriction digestion sites used during the donor vector assembly process. (**B**) Several key genome locus operations, such as epitope tagging, conditional regulation via 5′- and/or 3′-UTR manipulations and gene deletions are illustrated.

We opted for PCR-free transfer of these fragments to minimize the risk of introducing functionally deleterious mutations. We synthesized a gene block containing regions homologous to the fragments to be transferred using the Gibson assembly method ^22^, and pre-installed this into the parental pJAZZ linear vector to create pSwing (Fig. 1B and Supplementary Methods Fig. 2). We released both target fragments from the circular pSN372 and pSN1847 by ScaI and NotI double digestion, cut pSwing using SacII and used single pot, 3-piece Gibson assemblies to produce pSN372L and pSN1847L (Fig. 1B and Supplementary Methods Fig. 3). We screened bacterial colonies by restriction enzyme mapping to identify plasmids having the expected digestion pattern (Fig. 1C). These data show that large, pre-existing fragments containing AT-rich regions interspersed with regulatory components required for regulating *P. falciparum* gene expression are readily imported into this linear vector chassis.

### Linear vectors can be rescued into BACs that successfully transform *P. falciparum*

The linear vectors generated above must be converted into circular plasmids for stable episomal propagation or *Bxb1*-catalyzed chromosomal integration in *P. falciparum*. We rescued pSN372L and pSN1847L into BACs using an adapter plasmid (pAdapter) and a BAC recipient (BigBOB) as described ^21^. Fragments encoding components to be expressed in *P. falciparum* were released from pSN372L or pSN1847L by NotI/I-Sce1 restriction enzyme digestion, and assembled with pAdapter and BigBOB in single pot, 3-piece Gibson assembly reactions (Fig. 2A). We analyzed colonies containing the rescued pSN372R and pSN1847R BACs to confirm successful transfer of the pSN372L and pSN1847L fragments, respectively. Restriction digestion mapping showed that BACs with the expected topology were obtained (Fig. 2B). These data indicate that the linear vector-encoded sequence information critical for *P. falciparum* genetics studies can be readily rescued into BACs without the rearrangements frequently observed during construction of circular plasmids typically used for cloning AT-rich *P. falciparum* sequences.

### Regulatory components for controlling gene expression function predictably in BACs

Next, we sought to understand whether pSN372R and pSN1847R would: (1) successfully transform *P. falciparum*, both in episomal and chromosomally-integrated contexts; and (2) yield functional outcomes similar to those obtained using traditional *P. falciparum* expression vectors. We transfected pSN372R and pSN1847R with and without a plasmid encoding the *Mycobacterium Bxb1* integrase (pINT) ^15^ to achieve site-specific integration at the *cg6* locus in an NF54^attB^ line ^23^ or episomal maintenance, respectively. We selected parasites using Blasticidin S and monitored *Renilla* luciferase signal over the transfection. *Renilla* luciferase signal increased with the expected kinetics ^16^ for transfections expected to result in episomal BAC maintenance or *Bxb1*-mediated site-specific integration ^15^ of the BAC at the *cg6* locus (Fig. 2C). Taken together, these data indicate that BACs generated through this process successfully transform *P. falciparum* and the cassettes mediating *Renilla* luciferase and Blasticidin S deaminase transgene expression remain functionally intact.

We also wished to ensure that regulated gene expression mediated by the TetR and TetR–DOZI regulatory components hardwired into our linear vector and BAC frameworks was not compromised. Therefore, we measured anhydrotetracyline (aTc)-regulated expression of the FLuc reporter gene encoded by these constructs in parasites harboring chromosomally integrated pSN372R and pSN1847R. These data show that aTc regulates FLuc luciferase expression by eightfold (pSN372) and 115-fold (pSN1847) when a regular plasmid is integrated, and ~ sevenfold (pSN372R) and 145-fold (pSN1847R) when the rescued BACs are integrated (Fig. 2D). This degree of regulation is consistent with that observed in the context of the plasmids traditionally used in *P. falciparum* ^7,8^. Importantly, these data demonstrate that information needed to achieve gene expression and regulation outcomes in *P. falciparum* can be encoded on BAC constructs without compromising how these DNA parts function in the parasite.

### Modular linear vectors enable easily programmable and multi-functional designs

We generated several customizable base vectors pre-configured to achieve several key outcomes desired for functional genetics studies in *P. falciparum* (Fig. 3, Table 1 and Supplementary Fig. 1). We defined standardized architectures that collectively facilitate: promoter/5′-*UTR* swapping; N- and/or C-terminal epitope tagging; conditional regulation of gene expression; and genetic complementation by episomal or site-specific chromosomal integration. The components required for TetR aptamer-mediated regulation are hardwired into these base constructs. All variable parts needed to achieve locus-specific modification or transgene expression are modular, and we have enabled site-specific integration of any designed construct into the genome either through *Bxb1-*mediated *attB* × *attP* recombination, spontaneous single- and/or double-crossover integration or CRISPR/Cas9- and ZFN-mediated genome editing methods.

**Table 1:** Brief description of key plasmids described in this study.

Plasmid	Applications	Refs
pJAZZ	Original linear plasmid (Lucigen)	
pBigBOB	BAC used to rescue linear vectors (Fig. 2)	^21^
pAdapter	Used to rescue linear pJAZZ-derived vectors containing appropriate complementary sequences into a BAC (pBigBOB) context (Fig. 2)	^21^
pSN372L	- Contains a fragment released from a plasmid containing: a firefly luciferase reporter gene regulated via a single 5′UTR TetR aptamer; and a TetR_2A_RLuc_2A_BSD regulatory, monitoring and selection cassette	This work
pSN372R	- Same as pSN372L, but rescued into the BigBOB BAC	This work
pSN1847L	Contains a fragment released from a plasmid containing: a firefly luciferase reporter gene dually regulated by a single 5′UTR TetR aptamer and a 10 × TetR aptamer array in the 3′UTR; and a TetR-DOZI_2A_RLuc_2A_BSD regulatory, monitoring and selection cassette	This work
pSN1847R	- Same as pSN1847L, but rescued into the BigBOB BAC	This work
pSN154	- Conditional expression of epitope-tagged transgene from any promoter of choice - Can be configured such that regulation of transgene expression occurs via a single TetR aptamer within the 5′UTR, a 10 × TetR aptamer array in the 3′UTR or both; a TetR-DOZI_2A_RLuc_2A_BSD regulatory, monitoring and selection cassette is co-delivered -Linear plasmid can be rescued into the BigBOB BAC for either episomal maintenance or attB x attP site-specific integration using Bxb1 integrase	This work
pSN150	- Promoter swap with installation of a single regulatory TetR aptamer in the 5′UTR via CRISPR/Cas9 genome editing to facilitate conditional knockdown studies; a TetR_2A_RLuc_2A_BSD regulatory, monitoring and selection cassette is co-delivered in the process - Reconfigurable to enable CRISPR/Cas9-mediated gene knockout (validated herein)	This work
pSN053	- Installation of a regulatory 10 × TetR aptamer array in the 3′UTR by CRISPR/Cas9 genome editing to facilitate conditional knockdown studies; a TetR-DOZI_2A_RLuc_2A_BSD regulatory, monitoring and selection cassette is co-delivered in the process - Directly compatible with co-installation of a single TetR aptamer in the 5′UTR and a 10 × TetR aptamer array in the 3′UTR of a target gene - Reconfigurable to enable CRISPR/Cas9-mediated gene knockout (not tested)	This work
pSN054	- Installation of a regulatory 10 × TetR aptamer array in the 3′UTR by CRISPR/Cas9 genome editing to facilitate conditional knockdown studies; a TetR-DOZI_2A_RLuc_2A_BSD regulatory, monitoring and selection cassette is co-delivered in the process; the sgRNA cassette is chromosomally integrated in the process to provide a unique genetic barcode for the created cell line - Directly compatible with co-installation of a single TetR aptamer in the 5′UTR and a 10 × TetR aptamer array in the 3′UTR of a target gene - Reconfigurable to enable CRISPR/Cas9-mediated gene knockout (not tested)	This work

### Genetic complementation and transgene expression

pSN154 is designed to enable several outcomes, including gene complementation by either stable episome maintenance or *Bxb1* integrase-mediated chromosomal integration of rescued BACs via *attP* sites. Unique restriction sites modularize a region encoding a preinstalled T2A ‘skip peptide’ and HA epitope tag to allow synthesis of well-defined N-termini to either accommodate inclusion of leader peptides directing organelle-specific trafficking or optional epitope tags for protein detection (Fig. 3A). Similarly, C-terminal epitope tags (FLAG, c-Myc, HA) are preinstalled, and can be individually selected for inclusion in the encoded transgene. For regulated transgene expression, either a single and/or ten tandem TetR aptamers are preinstalled in the 5′- and 3′-*UTR*s, respectively. The 5′-UTR/promoter is modular to allow straightforward exchange for perturbing transgene expression timing and levels ^24,25^. A multi-cistronic regulatory module containing a TetR–DOZI_*T2A*_RLuc_*T2A*_Blasticidin S deaminase cassette using the T2A “skip” peptide is encoded on the same plasmid to permit regulated transgene expression (TetR–DOZI), quantitative monitoring of transfected parasites (RLuc), and positive selection of transformed parasites (Blasticidin S deaminase). This feature ensures that all constructs based on this design are compatible with any unmodified parental parasite strain, as all required regulatory components are co-delivered. Upon transgene insertion, this linear plasmid can be rescued into a BAC, and used to generate parasite lines in which the BAC is episomally maintained or chromosomally integrated (Supplementary Fig. 1A).

### Programmable donor vector contexts for genome editing

#### Design 1

pSN150 is designed to simultaneously facilitate promoter swapping and conditional regulation of target gene expression by a single TetR aptamer within a user-specified synthetic 5′-*UTR*. In this case, the regulatory module consists of a TetR_*T2A*_RLuc_*T2A*_blastisidin S deaminase cassette (Fig. 3). Modification of a target chromosomal locus is achieved by inserting the left (LHR) and right (RHR) homologous regions using the appropriate restriction sites (Fig. 3A and Supplementary Fig. 1B). A modular T2A ‘skip peptide’ and epitope tag (HA and c-Myc) region is included immediately downstream of the 5′-*UTR* aptamer. Translation most likely initiates using an ATG within the aptamer sequence, and this is expected to produce an 11 amino acid N-terminal leader peptide ^7,26^. We have previously shown this does not interfere with proper protein trafficking to organelles ^7^. Nevertheless, we have included the T2A feature to provide the option to force exclusion of this leader from the mature target protein. Similarly, the epitope tags can be electively retained or excluded from the mature protein. Overall, the modularity built into this region provides the flexibility to engineer the N-terminus of targeted proteins in a manner compatible with exploring their function irrespective of subcellular trafficking and compartmentalization. This design facilitates homology-directed repair of double strand DNA breaks induced by ZFNs or CRISPR/Cas9 at the target locus. For CRISPR/Cas9 editing, we have included a cassette for producing the required sgRNA under T7 promoter control ^13^. This cassette is easily modified to target a new locus by inserting the required targeting sequence via an I-PpoI/AflII site. AflII digestion is more efficient, and the preferred option.

#### Design 2

pSN053 and pSN054 (Fig. 3A) are intended to extend the possibilities for accessing native gene loci via manipulation of regions upstream, downstream and within a targeted gene. pSN053 and pSN054 are easily programmed to allow installation of regulatory aptamers within the 3′*UTR* only or both 5′- and 3′-*UTR*s to achieve aTc-dependent regulation via a TetR-DOZI-containing regulatory module. These options reflect our observation that TetR–DOZI, but not TetR, enables regulated gene expression via aptamers placed in the 3′-*UTR*, and that superior dynamic regulatory range is achieved when aptamers are dually positioned in the 5′- and 3′-*UTR*s ^8^. This configuration can be reasonably achieved while either preserving expression from the native promoter or swapping promoters, if desired (Supplementary Fig. 1C). More routinely, regulatory TetR aptamers can be introduced in the 3′-*UTR* of a target coding sequence (CDS) (Fig. 3A and Supplementary Fig. 1D). Modular N-terminal (HA) and C-terminal (FLAG, c-Myc and HA) epitope tags are preinstalled, allowing for flexible tag selection. An editable T7 promoter-driven sgRNA cassette to enable CRISPR/Cas9-mediated genome editing is also preinstalled. With pSN053, the sgRNA cassette is not integrated into the parasite’s chromosome during editing, while with pSN054, this cassette is chromosomally integrated and can be used as a barcode to uniquely identify parasite lines via a standardized PCR and either Sanger or Next Generation Sequencing methods. These linear plasmids or BAC-rescued versions can be used in Cas9-mediated genome editing applications. Rescued BACs are also suitable for 3′*UTR* modification achieved by single crossover integration.

### Increasing flexible options for genome editing in *P. falciparum*

**Figure 4:**
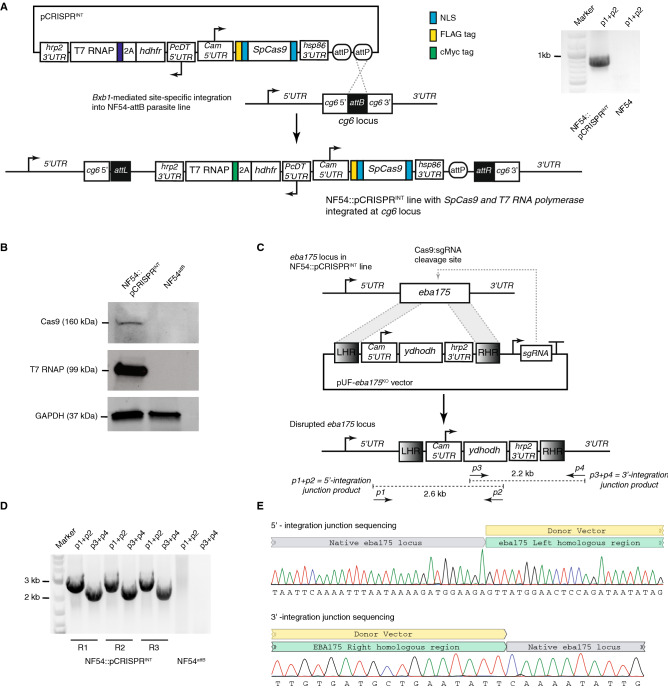
Validation of a pCRISPR plasmid reagent for CRISPR/Cas9-mediated genome engineering via transient or constitutive *Sp*Cas9 expression. (**A**) The pCRISPR plasmid allows constitutive expression of *Sp*Cas9 from the CAM 5′-*UTR*/hsp86 3′-*UTR* transcription unit. Through use of a T2A “skip peptide”, both the human dihydrofolate reductase (*hdhfr*) selectable marker and T7 RNA polymerase for sgRNA transcription are expressed from the *Pc*DT 5′-*UTR*/hrp2 3′-*UTR* transcription unit. The 2 × *attP* sites allow for *Bxb1*-mediated integration into a chromosomal *attB* site previously engineered into a host parasite strain. Successful integration of pCRISPR plasmid at the *cg6* locus in an NF54::pCRISPR parasite line was confirmed by PCR analysis. (**B**) Western blot analysis to detect expression of *Sp*Cas9 (anti-FLAG) and T7 RNA polymerase (anti-Myc) proteins by the NF54::pCRISPR parasite. (**C**) Schematic of the donor vector used to disrupt the dispensable *eba175* gene, and configuration of the *eba175* locus in the NF54::pCRISPR line pre- and post-editing. The donor vector contained left and right homologous regions from *eba175* flanking a selectable *ydhodh* expression cassette, and a T7 RNAP-transcribed cassette for expression of the sgRNA targeting *eba175*. (**D**) PCR analysis to detect the 5′- and 3′-integration junctions in edited lines using the primer pairs *p1* + *p2* and *p3* + *p4*, respectively. (**E**) Sanger sequencing data for the 5′- and 3′-integration junction PCR products obtained from the knockout transfection replicate, R3.

Our donor vector designs include the option for sgRNA production to facilitate Cas9-mediated genome editing. In our original implementation of CRISPR/Cas9 editing technology in *P. falciparum*, we used the orthogonal T7 RNA polymerase (T7 RNAP) to produce sgRNAs. We co-transfected parasites with a plasmid expressing *Sp*Cas9 and the sgRNA, and a donor vector expressing T7 RNAP and containing the required homology arms to repair Cas9-induced double strand breaks ^13^. We have streamlined this approach to allow simultaneous expression of *Sp*Cas9 and T7 RNAP from a single pCRISPR^*hdhfr*^ plasmid. T7 RNAP is produced along with the human DHFR selection marker using a T2A ‘skip peptide’ from a single expression cassette (Fig. 4A). pCRISPR^*hdhfr*^ contains an *attP* site to enable *Bxb1*-mediated integration into *attB* parasite lines. We have integrated pCRISPR^*hdhfr*^ into an NF54^*attB*^ strain ^23^ to create a clonal cell line stably expressing *Sp*Cas9 and T7 RNAP proteins (Fig. 4B), which can serve as a convenient background for the various described genome editing outcomes.

To validate that the NF54::pCRISPR line is competent for genome editing applications, we transfected this line with a homology-directed repair vector designed to disrupt the dispensable *eba175* invasion ligand through insertion of a DSM-1 selectable *ydhodh* expression cassette (Fig. 4C). We recovered viable parasites from independent triplicate transfections. In all cases, PCR and sequencing analyses confirmed that the expected insertion event into the *eba175* locus had been successfully achieved (Fig. 4D,E and Supplementary Fig. 2). Altogether, these new reagents increase the flexibility with which the *P. falciparum* genome can be edited either through co-transfection of donor vectors with the pCRISPR^*hdhfr*^ plasmid into a user-specified parasite strain, or by transfecting donor vectors into established pCRISPR^*hdhfr*^ cell lines.

### Linear plasmids facilitate donor vector assembly for engineering the *P. falciparum* genome to enable functional studies

To establish general applicability of this framework for functional genetics in *P. falciparum*, we sought next to demonstrate successful: (1) assembly of target constructs into the described linear vectors (pSN150 and pSN053/4, specifically) and successful rescue into BACs; and (2) use of these vectors to conveniently create genetically engineered parasites compatible with performing future biological studies aimed at studying parasite gene function in finer detail. We focused primarily on designs aimed at manipulating native gene loci.

- 1. Swapping native promoters for TetR aptamer-regulated synthetic promoters using CRISPR/Cas9 genome editing.

The timing and level of global gene expression during blood stage malaria parasite development is tightly regulated by both transcriptional and post-transcriptional mechanisms ^24,27–29^. The ability to perturb both of these gene expression parameters, therefore, can provide useful insights into how essential cell cycle, metabolic, and other biological processes are coordinated to ensure proper development. For example, previous work has shown that temporally ectopic expression of many parasite genes occurs when expression of CCR4-Associated Factor 1 (CAF1), a key regulator of mRNA metabolism, is disrupted. This resulted in severely dysregulated expression of genes involved in egress and invasion, and development of parasites in red blood cell stages was severely impaired ^30^.

Therefore, we sought to establish the technical feasibility of engineering several gene loci to replace native promoters with a non-cognate promoter/5′-*UTR* regulated by a single TetR aptamer. We selected five target genes for this proof-of-concept to sample both putatively essential and dispensable genes: choline kinase (CK; PF3D7_1401800); chloroquine resistance transporter (CRT; PF3D7_0709000); glycogen synthase kinase (GSK3; PF3D7_0312400); hexose transporter (HT; PF3D7_0204700); and thioredoxin reductase (TrxR; PF3D7_0923800). Except TrxR, these genes were considered to be likely essential in *P. falciparum* based on an insertional mutagenesis screen ^4^. In the related *P. berghei*, only HT and CRT were considered likely essential, while CK, GSK3 and TrxR were dispensable ^3^. These data suggest that CRT and HT are likely essential with highest confidence, and that knocking down their expression would more likely confer losses in parasite fitness and growth. Even with substantial knockdown, no functional outcome would be expected if CK, GSK3 and TrxR are dispensable in accordance with the *P. berghei* data. Notably, the editing efficiency determined by limiting dilution cloning, was comparable between the HT, GSK3 and TrxR, indicating that low representation of edited parasites was not the reason for this observation (Supplementary Fig. 3). On the other hand, if essential as suggested by the piggyBAC screen, even modest knockdown levels could result in loss of parasite fitness.

**Figure 5:**
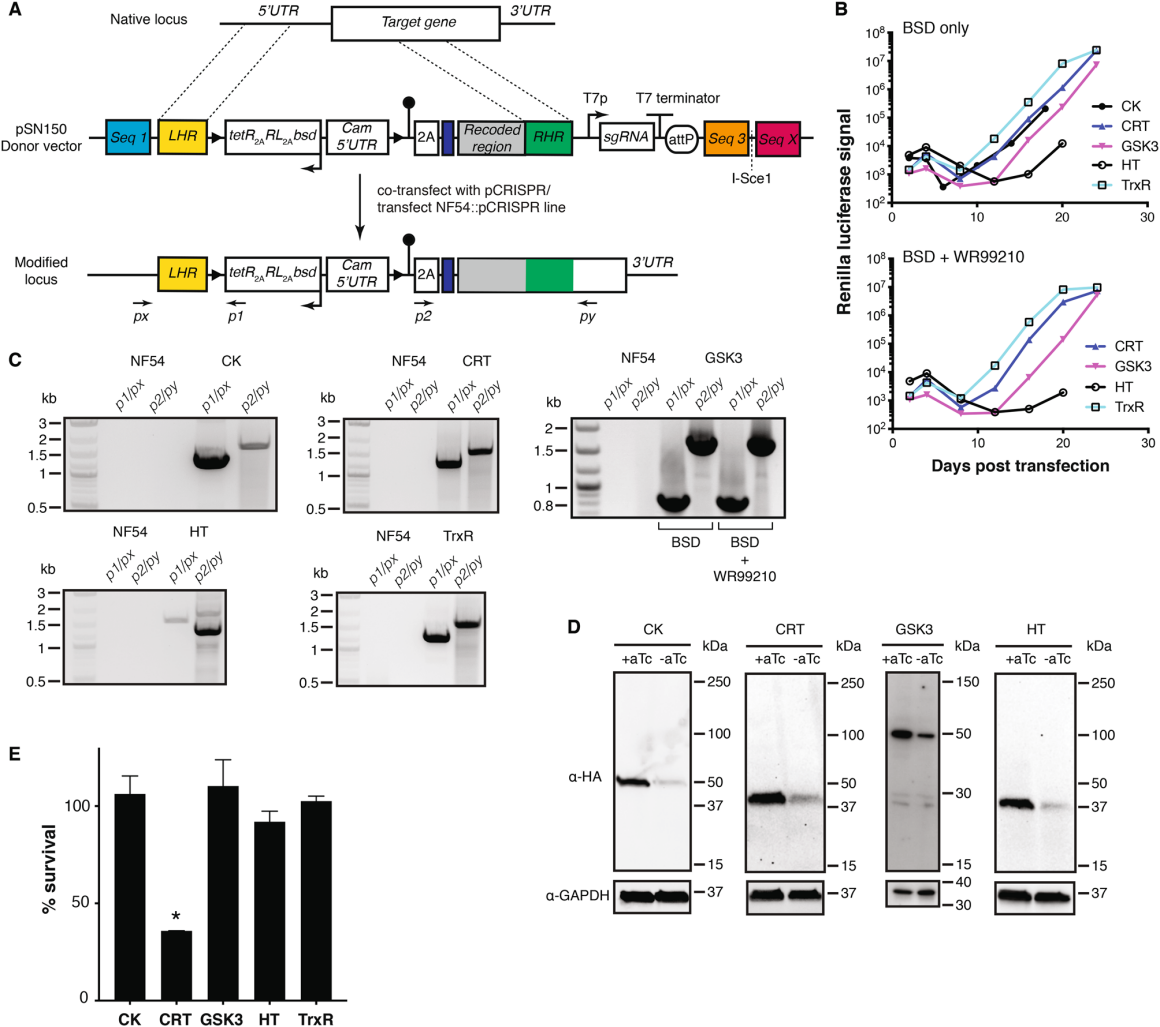
Manipulating *5′-UTRs* of native loci using BAC-rescued or pSN150 donor vectors and CRISPR/Cas9 genome editing. (**A**) Generalized schematic of CRISPR/Cas9-mediated modification of a target locus to install a synthetic 5′UTR regulated by a single TetR-binding aptamer, an N-terminal HA tag and an expression cassette producing TetR, *Renilla* luciferase (RL) and the Blasticidin S deaminase selection marker. Engineered parasites were generated by either co-transfecting donor vectors with the pCRISPR plasmid or transfecting into the NF54::pCRISPR line described in Fig. 4. (**B**) Successful generation of stable parasite lines was monitored via *Renilla* luciferase levels, under conditions where only the donor vector was positively selected (BSD) or both donor vector and pCRISPR plasmids were selected (BSD + WR99210). (**C**) gDNA extracted from transgenic parasites analyzed by PCR demonstrated formation of the expected *5′*- and *3′*- integration junctions at each targeted locus. Expected junctional PCR product sizes (*5′*, *3′*): *CK* (1.3 kb, 1.7 kb)*; CRT* (1.2 kb, 1.6 kb); *GSK3* (0.84 kb, 1.68 kb); *HT* (1.6 kb,1.6 kb); and *TrxR* (1.3 kb,1.7 kb). Marker = 1 kb Plus DNA ladder (New England Biolabs). Full gel images are included in Supplementary Fig. 4A–E. (**D**) Western blot analysis of target protein expression under ± aTc conditions. Expected molecular weights of the HA-tagged proteins are: CK = 53.7 kDa; CRT = 50.2 kDa; GSK = 51.6 kDa; and HT = 57.9 kDa. *Note*: HT migrates faster than its expected molecular weight, but identically when detected by an N- and C-terminal tag (Fig. 7). Full gel images are included in Supplementary Fig. 4F–I. (**E**) Normalized *Renilla* luciferase levels or SYBR Green I staining (GSK3 line only) for parasites grown in the absence (0 nM) or presence (50 nM) aTc for 72 h. Data represent the mean of *n* = 3 ± s.e.m for CK, CRT, HT, and TrxR, and *n* = 2 ± s.e.m for GSK3. * *p* ≤ 0.05 by Student’s t-test.

We used a 625 bp fragment derived from the *P. falciparum* calmodulin (Cam) promoter ^31^ modified by a single, regulatory TetR aptamer (${Cam}_{625 bp}^{5{^{\prime}}apt}$) as the non-cognate, synthetic promoter/5′-*UTR* to replace the cognate promoter/5′-*UTR*s in these experiments. Relatively few instances of functional 5′-*UTR* manipulation in *Plasmodium* blood stages have been reported ^7,32^. This reflects a combination of: (1) technical difficulties associated with 5′-*UTR* engineering using previous approaches; and (2) the challenge of ensuring quantitatively adequate expression of essential proteins to allow parasite survival at baseline, and then triggering sufficient protein depletion such that levels fall below the functional threshold. Using pSN150, we designed and assembled constructs to exchange the native promoter, and install an HA tag at the expected N-terminus of each target protein (Fig. 5A). For proteins with signal peptides, it is possible to create designs in which an epitope tag is positioned downstream of the expected signal peptide cleavage site. However, there is no guarantee such an engineered N-terminus would be correctly processed to permit correct subcellular trafficking of the tagged, mature protein. We note that all proteins evaluated here do not have predicted N-terminal signal/ trafficking peptides. Upstream and downstream homologous regions needed for editing the intended locus were PCR amplified from within the native 5′-*UTR* and target gene, respectively. To ensure that repair is irreversible and simultaneously preserve the option to use the same donor vector with alternative sgRNAs, we synthesized recoded DNA segments beginning at the ATG of the target coding sequence through a region containing several candidate Cas9-sgRNA target sites. It is noted here that recodonization may carry the risk of eliminating or introducing sequence information that may adversely impact regulation of gene expression. We have no objective experimental data to qualify this risk, but include this statement as a note of awareness to users.

We co-transfected NF54^attB^ parasites with pSN150 donor BACs targeting CK, CRT, GSK3, HT and TrxR with pCRISPR^*hdhfr*^ in the presence of aTc to generate edited lines. We selected for either the donor BAC only (BSD) or both donor BAC and pCRISPR (BSD + WR99210), and successfully recovered transformed parasites using both selection protocols (Fig. 5B). This confirmed suitability of pSN150-derived BACs for generating transgenic parasites, and adequate production of putatively essential CRT and HT proteins using a non-cognate synthetic promoter. Using PCR and sequencing, we verified that parasites were edited as expected. For all target genes, independently of whether the pCRISPR^*hdhfr*^ plasmid was co-selected, we detected the expected integration events in transgenic lines, but not the parental NF45^*attB*^ (Fig. 5C, Supplementary Fig. 4A–E). Western blot analysis revealed aTc-dependent regulation of CK, CRT, GSK3 and HT protein expression (Fig. 5D, Supplementary Fig. 4F–I). TrxR could not be detected, even though DNA sequencing confirmed the epitope tag was in-frame with the TrxR coding sequence. This observation is consistent with the previous studies showing that two isoforms of TrxR are produced: a cytosolic form initiated from an internal, alternate translation initiation site; and a mitochondrial form resulting from N-terminal processing of the expected full-length protein ^33^. Therefore, it is likely that mature TrxR produced by the parasite excludes the N-terminal epitope tag.

We compared aTc-dependent growth of these nonclonal engineered parasites. No significant aTc-dependent difference in relative growth was observed for the engineered CK, GSK3 and TrxR lines, but growth of the engineered CRT line decreased by ~ 60% after 72 h under knockdown conditions (Fig. 5E). For the engineered HT line grown in standard RPMI with 2 mg/mL glucose, no aTc-dependence in growth was detected. As HT mediates glucose transport, we also evaluated growth under more limiting glucose conditions. In 0.2 mg/mL glucose there was ~ 50% reduction in growth after 72 h in the absence of aTc (Supplementary Fig. 5). These data show that, consistent with their expected essential functions, conditional knockdown of CRT and HT expression levels produced measurable reductions in parasite growth. In contrast, no such growth deficits were detected with the potentially dispensable CK, GSK3 and TrxR knockdown lines.

**Figure 6:**
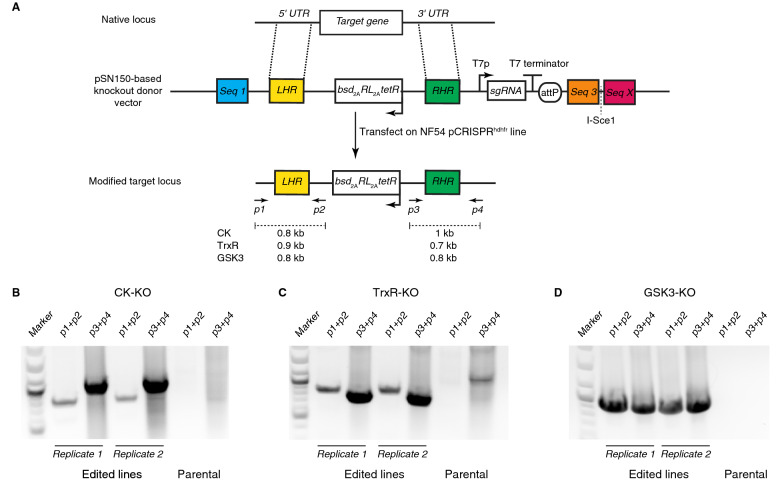
Linear pSN150 can be used for efficient CRISPR/Cas9-mediated deletion of non-essential *P. falciparum* loci. (**A**) Schematic of a pSN150 vector configured to delete a target locus via homology-directed repair after Cas9-induced cleavage mediated by a sgRNA transcribed from the same vector. (**B-D**) PCR analysis to detect the 5′- and 3′-integration junctions diagnostic of successfully edited parasite lines using the primer pairs *p1* + *p2* and *p3* + *p4*, respectively. *p1* and *p4* primers were selected to recognize the CK, TrxR and GSK3 loci being targeted, while *p2* and *p3* are common primers that bind, respectively, within the *hrp2* 3′-*UTR* and hsp86 5′-*UTR* common to all pSN150-derived knockout vectors.

Given the discordance between piggyBAC mutagenesis data from *P. falciparum* and PlasmoGEM knockout data from *P. berghei* with respect to CK and GSK3 essentiality, we used the pSN150 framework to generate knockouts in *P. falciparum*. Beginning with the vectors used to modify the 5′-*UTR*s of CK and GSK3, we preserved the *bsd* selection cassette, left homologous region and sgRNA cassette, but replaced the fragment containing the synthetic promoter through to the original right homologous region with a new right homologous region overlapping with the 3′-*UTR* of the targeted locus (Fig. 6A). We included a similarly designed construct for TrxR as a control for a non-essential gene, as direct transposon insertion into the coding sequence at this locus was detected in the *piggyBAC* screen ^4^. We transfected linear plasmids, each in duplicate, into the NF54::pCRISPR^*hdhfr*^ line under BSD selection pressure, since successful homology directed repair of the target locus will result in chromosomal integration of the BSD resistance to facilitate selection. In all instances, we recovered BSD-resistant, RLuc positive parasites post-transfection. PCR and sequencing analysis of genomic DNA isolated from these parasites revealed that successful disruption of the targeted locus had been achieved in all instances (Fig. 6B–D). Thus, consistent with the *P. berghei* data, our findings indicate that CK, GSK3 and TrxR are dispensable for *P. falciparum* asexual blood stage growth.

Altogether, these data indicate that genome engineering using easily-designed pSN150-based donor vectors and CRISPR/Cas9 methods can be used to efficiently reconfigure promoter/5′-*UTR* regions in their native chromosomal context. Appropriately selected synthetic promoters can also be substituted for native promoters to achieve regulated and functionally informative perturbation of essential target protein expression. Furthermore, pSN150-based donor vectors can be conveniently reconfigured to create targeted gene deletion vectors that can be used in conjunction with parasite cell lines stably expressing Cas9 to evaluate gene essentiality. These easy-to-implement and high-resolution gene deletion manipulations are complementary to recently-described genome-scale approaches ^3–5^ as tools to rigorously validate gene essentiality, as highlighted here for CK and GSK3. The potential to scale up production of these vectors provides opportunities for use in primary screens implemented with high gene-targeting efficiency and specificity, while introducing well-defined loss-of-function genetic changes. Such desired outcomes may not be consistently attainable in random insertional mutagenesis screens, especially if full saturation is not achieved. Altogether, the combined use of these various approaches will be crucial for most accurately characterizing essential gene function in *P. falciparum*.

- 2. Native allele modification to install C-terminal epitope tags and regulatory TetR aptamers in 3′-*UTR* and both 5′- and 3′-*UTR*s by CRISPR/Cas9 genome editing.

Modifying the 3′-*UTR* of genes in their chromosomal contexts permits insertion of epitope tags and regulatory elements while preserving native timing and similar expression levels associated with the endogenous promoter. This is desirable in functional studies requiring perturbations in protein levels that more precisely match physiological expression timing so as to facilitate elucidation of biological function.

**Figure 7:**
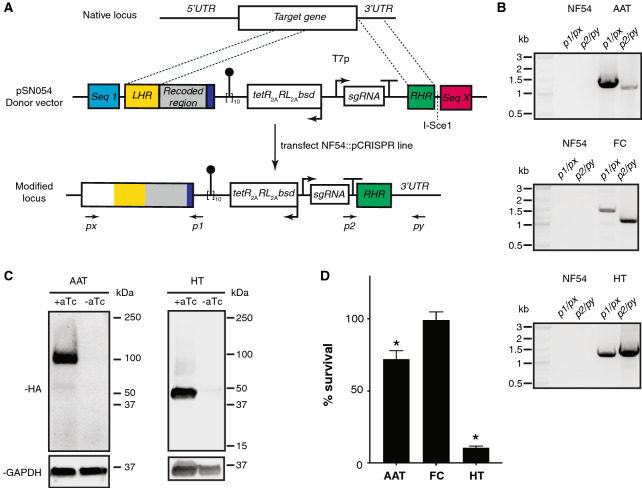
Manipulating the 3*′-UTRs* of native loci using linear pSN054 donor vectors and CRISPR/Cas9 genome editing. (**A**) Generalized schematic of CRISPR/Cas9-mediated editing of a target locus to install C-terminal 2x-HA epitope tags, a 10 × TetR aptamer array, and a TetR-DOZI, *Renilla* luciferase and Blasticidin S deaminase expression cassette. Donor vectors were transformed into the NF54::pCRISPR parasite line to modify targeted loci. (**B**) gDNA extracted from transgenic parasites analyzed by PCR demonstrated formation of the expected 5*′*- and 3*′*- integration junctions at each targeted locus. Expected junctional PCR product sizes (5*′*, 3*′*): *AAT*, putative amino acid transporter (1.4 kb, 1.1 kb); FC, ferrochelatase (1.5 kb, 1.2 kb); and HT, hexose transporter (1.3 kb, 1.4 kb). Marker = 1 kb Plus DNA ladder (New England Biolabs). Full gel images are included in Supplementary Fig. 6A–C. (**C**) Western blot analysis of target protein expression under ± aTc conditions. Expected molecular weights of the 2 × HA-tagged proteins are *AAT* = 137.6 kDa and HT = 60.5 kDa. No HA-tagged FC protein was detected, even after multiple attempts. *Note*: AAT and HT migrate faster than expected based on their molecular weights; however, N- and C-terminal tagged HT migrate identically (Fig. 5). Full gel images are included in Supplementary Fig. 6D–E. (**D**) Normalized *Renilla* luciferase levels for parasites grown in 0 nM or 50 nM aTc for 72 h. Data represent the mean of *n* = 3 ± s.e.m. * *p* ≤ 0.05 by Student’s t-test.

To achieve this, we have used pSN053 and/or pSN054 together with CRISPR/Cas9 genome editing to readily modify diverse loci (Fig. 7A and Supplementary Fig. 1C,D). Here, we show this by targeting the hexose transporter (HT; PF3D7_0204700), ferrochelatase (FC; PF3D7_1364900) and a putative amino acid transporter (AAT; PF3D7_0209600) using pSN054-based donor vectors. Once sgRNAs targeting a site near the 3′-*UTR* of these genes were selected, left homologous regions together with a recoded region overlapping the sgRNA target site and right homologous regions corresponding to part of the 3′-*UTR* were used to create donor vectors. These donors were transfected into the NF54::pCRISPR line to generate edited lines that were verified for appropriate integration at the target site by junctional PCR and sequencing of the amplified PCR product (Fig. 7B, Supplementary Fig. 6A–C). Western blot analysis of the AAT and HT lines showed aTc-dependent regulation of protein expression (Fig. 7C, Supplementary Fig. 6D–E). Despite in-frame integration of the epitope tag with FC, no tagged FC protein was detected under induced conditions. One possible explanation is that this protein is not highly expressed, which could be consistent with the low transcript levels observed across the intraerythrocytic developmental cycle (PlasmoDB.org: PF3D7_1364900).

Inducing knockdown of HT and AAT, and putatively FC produced a range of responses in parasite growth. For HT, we detected a dramatic loss in parasite viability upon HT depletion, consistent with an essential role. This effect occurred in standard high glucose concentration (2 mg/mL) media, in contrast to the relatively more subtle glucose concentration-dependent growth phenotype observed with the regulated, synthetic promoter (Fig. 7D and Supplementary Fig. 5). This is consistent with the greater degree of protein knockdown achieved using a 10 × aptamer array within the 3′-*UTR* (Fig. 7C) compared to a single aptamer regulating the synthetic 5′-*UTR* (Fig. 5D). For AAT, we observed an ~ 30% decrease in parasite viability (Fig. 7D) after a single replication cycle during which verifiable depletion of detectable protein occurred (Fig. 7C). Interestingly, both *piggyBAC* mutagenesis ^4^ and *P. berghei* knockout screens ^3^ classify this gene as essential, although the knockdown studies here suggest that acute loss-of-function of this gene induces a fitness cost. Putative FC depletion through aTc removal did not result in any detectable change in parasite growth, which would be consistent with the non-essentiality of this protein and de novo heme biosynthesis during blood stage parasite development ^3,4,34–36^.

Even broader utility of pSN053/054 for enabling in-depth functional studies of diverse parasite genes is underscored by their application in elucidating roles for the claudin-like apicomplexan microneme protein (CLAMP) ligand in red blood cell invasion ^5^, aspartate proteases Plasmepsin IX and X in red blood cell invasion and egress ^37^, and FtsH1 and ATG8 in apicoplast biogenesis/maintenance ^38,39^. In some instances, it can be desirable to achieve even more stringently regulated knockdown to study biological function using a dual aptamer configuration ^8^. This is readily attained via pSN053/pSN054 (Supplementary Fig. 1C), as illustrated during work defining new, essential roles for the integral membrane protein, EXP2 ^40^ and Plasmepsin V protease ^41^ beyond their previously described roles in protein export from the parasite to the red blood cell compartment ^42,43^.

## Conclusions

We describe a flexible plasmid toolkit that improves the ease with which genome manipulation of the less genetically tractable but devastating human malarial parasite, *Plasmodium falciparum*, can be engineered to study gene function. This toolkit leverages the significantly increased efficiency linear plasmids afford in manipulating large and complex AT-rich DNA sequences without the undesirable deletions and rearrangements frequently encountered with typically-used circular bacterial plasmids. We have emphasized modular designs to allow facile and standardized vector configuration to address a broad variety of functional applications, including gene complementation, conditional regulation of gene expression, and gene deletions. We have ensured direct compatibility with CRISPR/Cas9 genome engineering by hardwiring an easily reprogrammed guide RNA production module into base donor vectors. We have also validated a pCRISPR plasmid that can be used to transiently or constitutively produce Cas9 when either co-transfected as a helper plasmid or stably integrated into a desired background strain, respectively. Importantly, any construct assembled in a linear vector can be ‘rescued’ with high efficiency to a circular BAC, while keeping AT-rich and repetitive sequences intact. In *P. falciparum*, only circular DNA is stably maintained episomally, and integrase-mediated site-specific integration is efficiently achieved from circular DNA. Therefore, this feature extends the approaches accessible for evaluating gene function in human malarial parasites. Altogether, we anticipate the toolkit described here will enable more technically robust strategies for high-resolution genome manipulation to improve our fundamental understanding of malaria parasite biology and enable applications that can enhance discovery of novel antimalarial therapeutics and disease prevention strategies.

## Methods

### Molecular biology and plasmid construction

The methods used to assemble the vectors reported in this study are included as Supplementary Methods. These are described in step-by-step detail to allow users with basic knowledge of molecular cloning to easily build constructs in the pSN150, pSN154 and pSN053/054 contexts.

### Parasite culturing and transfection

*P. falciparum* strain 3D7 parasites were grown under 5% O_2_ and 5% CO_2_ in RPMI-1640 media supplemented with 5 g/L Albumax II (Life Technologies), 2 g/L sodium bicarbonate, 25 mM HEPES pH 7.4 (pH adjusted with potassium hydroxide), 1 mM hypoxanthine and 50 mg/L gentamicin. Human red blood cells from a commercial supplier (Research Blood Components, Watertown, MA) were used for parasite cell culture. Transfections were performed using the red blood cell preloading method as described previously ^19^. Briefly, 50–100 μg of purified plasmid DNA were mixed with human red blood cells in 0.2 cm cuvettes and subjected to 8 square wave electroporation pulses of 365 V for 1 ms each, separated by 0.1 s. The DNA preloaded red blood cells were inoculated with schizont-stage parasites (e.g. NF54^attB^, NF54::pCRISPR) to achieve starting parasitemias ≤ 1% in RPMI 1640 Complete media. Beginning 4 days post-transfection, cultures were selected with either 2.5 µg/mL Blasticidin (RPI Corp, B12150-0.1) or a combination of 2.5 µg/mL Blasticidin and 2.5 nM WR99210 (Jacobus Pharmaceuticals). In knockout experiments performed on the NF54::pCRISPR background, parasites were selected with 1.5 µM DSM1 (MR4) only beginning on Day 4 post-transfection. For creating knockdown lines using pSN150 and pSN053/54 vectors, 500 nM anhydrotetracycline (aTc; Sigma-Aldrich, 37,919) was included at the beginning of transfections and maintained throughout. Transfection progress was monitored using Giemsa-stained smears and *Renilla* luciferase measurements. High purity genomic DNA was isolated using the Blood & Cell Culture DNA Mini Kit (Qiagen 13323) according to the manufacturer’s instructions. Cultured cells were lysed under denaturing conditions, and Proteinase K was added to degrade proteins. The suspension was loaded onto a sterile Qiagen Genomic tip. After the recommended incubation period, the sample was centrifuged and the supernatant discarded. Following a wash step, the silica membrane was transferred to a new snap cap, safe lock microcentrifuge tube and distilled, deionized water or TE buffer used to elute DNA. Modification of the target locus was determined by PCR using appropriate locus-specific primers (Supplementary Methods).

### Western blotting analysis

Western blotting for *Sp*Cas9 and T7 RNAP was described previously ^13^. To determine regulation of target protein expression by the TetR- or TetR-DOZI-aptamer system, parasites cultured with 0 nM or 50 nM aTc for 72 h were saponin-lysed, washed with 1 × PBS, and proteins solubilized in lysis buffer containing 4% sodium dodecyl sulfate (SDS) and 0.5% Triton X-114 in 1 × PBS. Protein extracts were mixed with loading buffer containing SDS and dithiothreitol (DTT) and loaded onto Mini-PROTEAN TGX Precast Gels (4–15% gradient; Bio-Rad 4,561,084) in Tris–Glycine buffer. After separation by polyacrylamide gel electrophoresis (PAGE), proteins were transferred to a polyvinylidene fluoride (PVDF) membrane using the Mini Trans-Blot Electrophoretic Transfer Cell system (Bio-Rad) per the manufacturer’s instructions and blocked with 100 mg/mL skim milk in 1 × TBS/Tween 20. PVDF membrane-bound proteins were first probed with mouse anti-HA (1:3000; Sigma, H3663) and rabbit anti-GAPDH (1:5000; Abcam, AB9485) primary antibodies and anti-mouse (1:5000; Thermo Fisher Scientific, 62–6520) and anti-rabbit (1:5000; Cell Signaling, 7074S) horseradish peroxidase (HRP)-conjugated secondary antibodies. Following incubation in SuperSignal West Pico Chemiluminescent substrate (Thermo Fisher Scientific, PI34080), protein blots were imaged and analysed using the ChemiDoc MP System and Image Lab 5.2.0 (Bio-Rad).

### Luciferase and quantitative growth assays

Luciferase assays to track transfection progress and to measure aTc-dependent regulation of a firefly reporter were performed as previously described ^8,16^ using the Dual-Luciferase Reporter Assay System (Promega, E1910), Renilla Luciferase Assay System (Promega, E2810) or Renilla-Glo Luciferase Assay System (Promega E2750) per manufacturer’s protocols. Quantitative growth assays were performed in 96-well U-bottom plates (Corning 62406-121) using synchronous ring-stage parasites set up in triplicate and cultured in the 0 nM or 50 nM aTc. Relative growth was determined using luciferase levels measured at 0 h (initial setup values) and after 72 h using the GloMax Discover Multimode Microplate Reader (Promega). Luminescence values were normalized to samples treated with a lethal dose of chloroquine (200 nM) as no growth. Data was analyzed using GraphPad Prism (version 8; GraphPad Software). For GSK3 knockdown experiments, parasite growth was determined by FACS analysis using an Accuri Flow Cytometer (BD Biosciences). Parasites were stained for nucleic acid content with 1 μM SYTO 61 (Life Technologies) and analyzed in the FL4 signal channel to determine the fraction of parasitized red blood cells (parasitemia).

For comparing the functional HT regulation achieved using the pSN150 and pSN054 configurations, we measured the expansion of synchronous ring stage parasites after a 72 h period of growth. Assays were set up at various initial parasitemias (0.1%, 0.5% and 1.5%) in various glucose (0.2, 0.8, 1.4 and 2.0 mg/mL) and aTc (0, 1, 3 and 50 nM) concentrations. RPMI 1640 without glucose (US Biological, R8999-13) was used as the base medium, with supplementation as needed with glucose and aTc.

## Supplementary information

Supplementary information.
