# Supplementary material for: An integrated platform for genome engineering and gene expression perturbation in Plasmodium falciparum
Source: Sci Rep. 2021 Jan 11;11:342. doi: 10.1038/s41598-020-77644-4 (PMC7801740; doi:10.1038/s41598-020-77644-4)
Supplement: Supplementary file 1 — Supplementary information. [file 41598_2020_77644_MOESM1_ESM.pdf]

An integrated platform for genome engineering and gene expression perturbation in *Plasmodium falciparum*

Armiyaw S. Nasamu[1], Alejandra Falla[1], Charisse Flerida A. Pasaje[1], Bridget A. Wall[1], Jeffrey C. Wagner[1], Suresh M. Ganesan[1], Stephen J. Goldfless[1] and Jacquin C. Niles[1]

[1]Department of Biological Engineering, Massachusetts Institute of Technology, Cambridge MA 02139

Correspondence:

Jacquin C. Niles

Department of Biological Engineering

Massachusetts Institute of Technology

77 Massachusetts Avenue

Room 56-341B

Cambridge MA 02139

Email: jcniles@mit.edu

Telephone: (617) 324-3701

Fax: (617) 324-5698

SUPPLEMENTARY METHODS

Materials

pJAZZ-OC Not I Vector (Lucigen, Catalog # 43024)
BigEasy-TSA Electrocompetent Cells (Lucigen, Catalog # 60224)
BAC-Optimized Replicator v2.0 Electrocompetent cells (Lucigen, Catalog # 60210)
2x Gibson Assembly Master Mix (NEB, Catalog #E5510)
DNA Polymerase I, Large (Klenow) Fragment (New England Biolabs, Catalog# M0210)
Restriction enzymes were purchased from New England Biolabs, unless otherwise stated

Section I. Creating base linear vectors while incorporating components from traditional circular plasmids.

The pJAZZ-OC vector (Lucigen) was converted into the various pSN configurations as summarized in the scheme below (*Supplementary Methods Figure 1*).

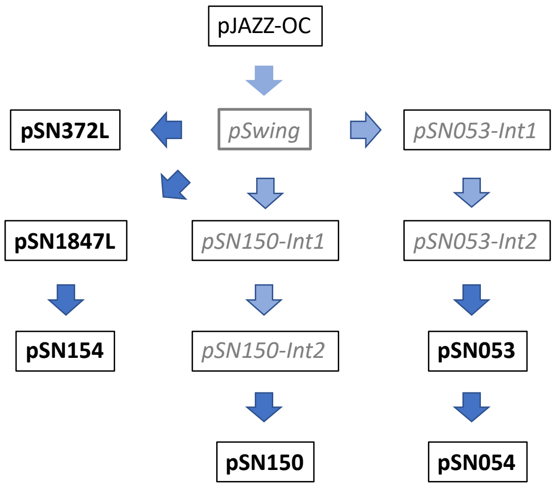

Supplementary Methods Figure 1. A flow chart summarizing conversion of pJAZZ-OC into the various linear pSN base vectors. Grey italics and light blue arrows denote intermediate steps en route to final plasmids (boldface and darker blue arrows).

pSwing construction

pJAZZ-OC Not I Vector (200 ng) was digested with NotI at 37 °C for 90 min and the reaction heat inactivated at 65 °C for 20 min. The pSwing gblock (*Supplementary Methods Figure 2*) containing the unique sequences 1, 3 and X (SEQ1, SEQ3, SEQX) and FseI, SacII and I-SceI restriction sites was obtained from IDT. The digested vector (20 ng) and gblock (20 ng) were mixed with an equal volume of 2x Gibson Assembly Master Mix and incubated at 50 ºC for 1 h to assemble an intermediate vector, pSwing. Big Easy TSA cells were transformed with 1 µL Gibson reaction mixture, and plated on LB-agar with chloramphenicol (34 µg/mL) and incubated overnight at 30 ºC. Selected colonies were grown overnight in liquid LB supplemented with chloramphenicol (34 µg/mL), mini-prepped and verified by restriction digestion and sequencing.

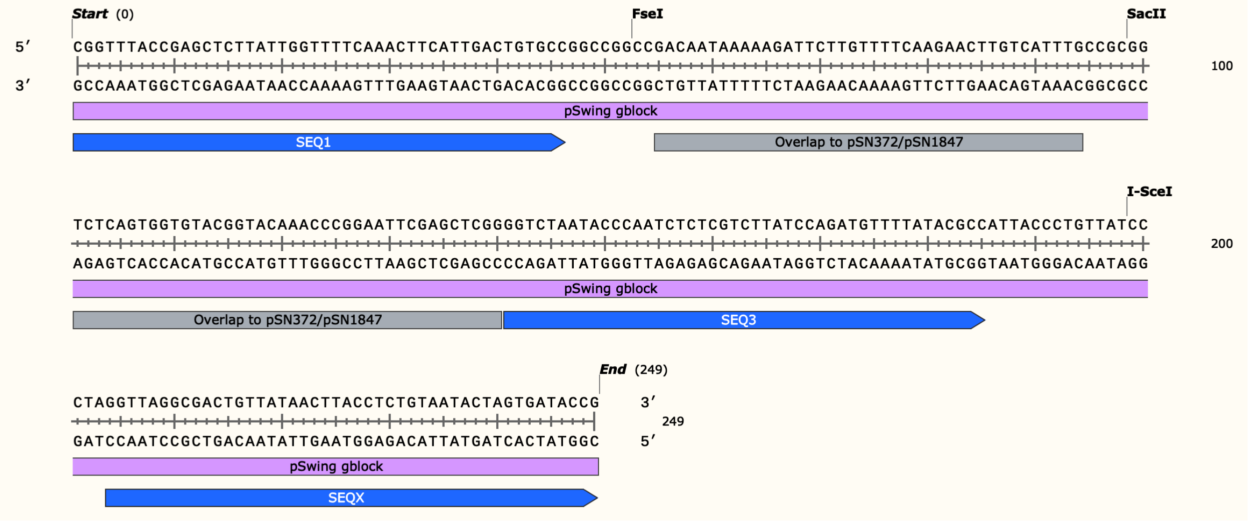

Supplementary Methods Figure 2. pSwing gblock sequence map and features.

pSN372L and pSN1847L construction

Plasmids pSG372.3 and pMG56 (Ganesan *et al*, 2016) were first modified to produce pSN372 and pSN1847. pSN372 was made by inserting via Gibson assembly: (1) a loxP-containing DNA fragment (<u>tctattattaaataaatttaatgga</u>**ataacttcgtatagcatacattatacgaagttat**<u>ccggtttagccctcccacacataac</u>; loxP site in bold font and Gibson overlap underlined) via an AgeI site upstream of BSD; and (2) a second loxP-containing fragment (<u>atacctaatagaaatatatcttaag</u>**ataacttcgtatagcatacattatacgaagttat***aataaatacctaatacaatccaggc*<u>caatccaggcagagaaaggtcgatac</u>; loxP site in bold, spacer in italics and Gibson overlap underlined) via an AflII site downstream of the Cam promoter. pSN1847 was created by performing the second step above on pMG56. Linear pSN372L and pSN1847L were created from their respective pSN372 and pSN1847 circular plasmids. pSwing (200 ng) was digested with SacII at 37 °C for 1.5 h, followed by heat inactivation of enzyme at 65 °C for 20 min. Inserts from pSN372 and pSN1847 were released by ScaI+NotI double digestion at 37 °C for 1 h, followed by heat inactivation of enzymes at 80 °C for 20 min. Released inserts (20 ng) were each mixed with SacII-digested pSwing (20 ng) and an equal volume 2x Gibson Assembly Master Mix, and incubated at 50 ºC for 1 h. Big Easy TSA cells were transformed and colonies analyzed as above to isolate pSN372L and pSN1847L (*Supplementary Methods Figure 3A,B*). Mini-prepped plasmids were analyzed for proper assembly by NotI and FseI+AflII (pSN372L) and Aflll+NotI (pSN1847L) digestions.

A. pSN372L

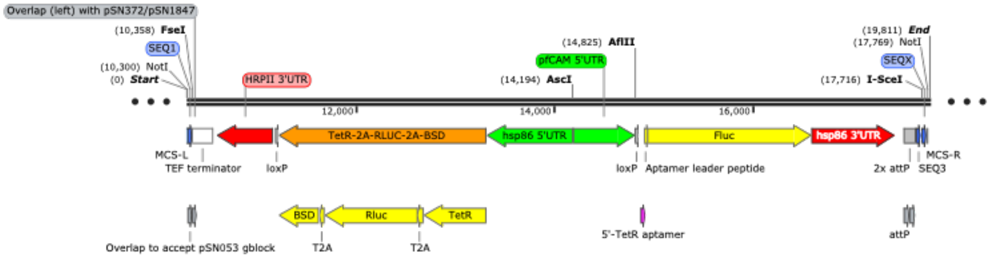

B. pSN1847L

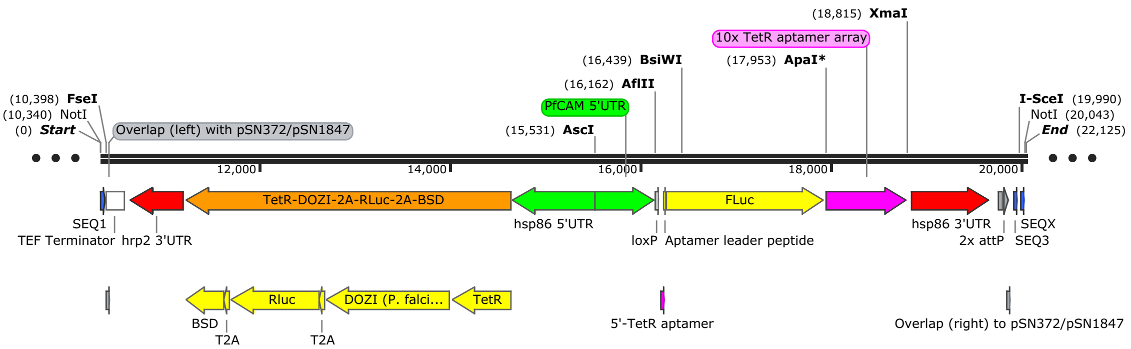

Supplementary Methods Figure 3. Overview map of (A) pSN372L and (B) pSN1847L.

pSN154 construction

The *Firefly* luciferase gene in pSN1847L was removed by AflII+ApaI digestion and replaced with the pSN154-gblock to obtain pSN154 (*Supplementary Methods Figure 4A,B*).

A. pSN154 gblock

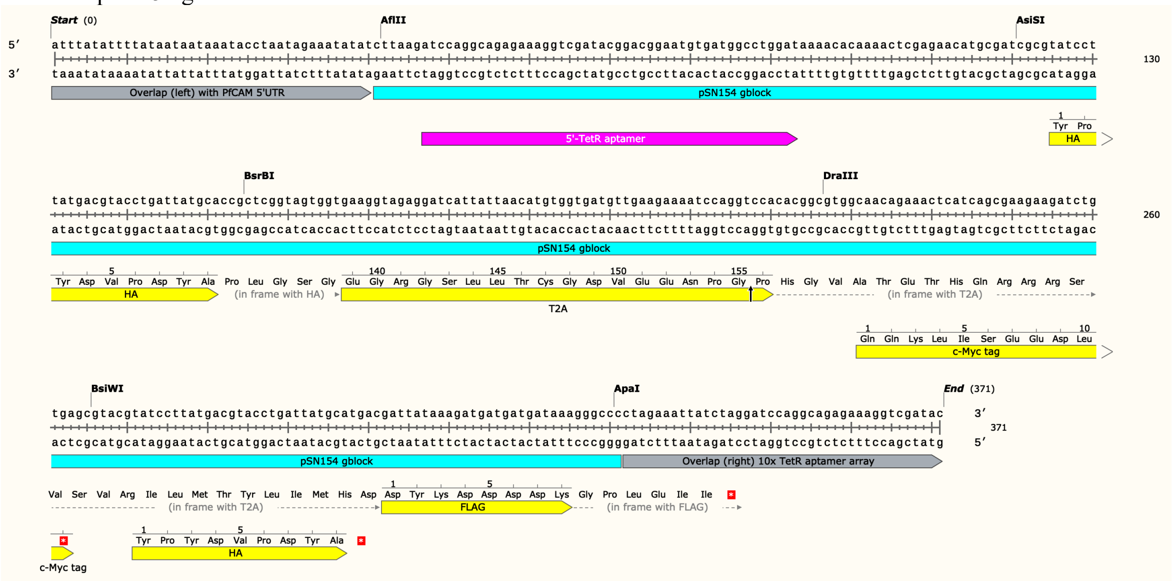

B. pSN154

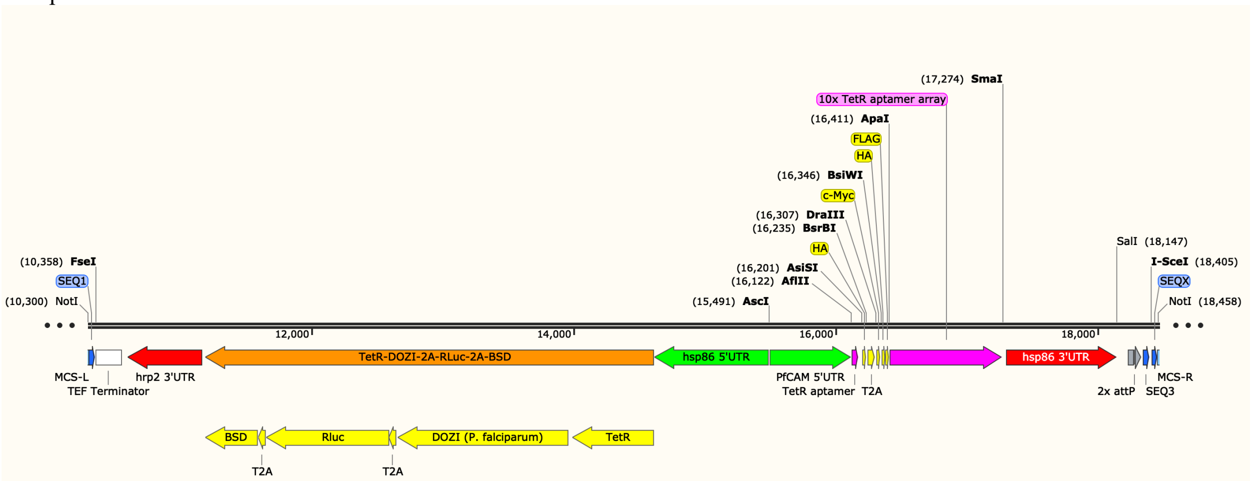

Supplementary Methods Figure 4. Overview of pSN154 construction. (A) pSN154-gblock sequence and features. (B) Map of final pSN154 plasmid.

pSN150 construction

The pSN150-gblock (IDT) was cloned by Gibson assembly into FseI+SacII-digested pSwing to create pSN150-Int1 (*Supplementary Methods Figure 5A,B*). A SEQ1-(FseI)-*hrp2* 3'UTR-TetR-2A-RLuc-2A-BSD-*hsp86* 5'UTR-(AscI)-*PfCAM* 5'UTR-loxP-TetR aptamer fragment released by NotI+XhoI digestion of pSN372L was cloned by Gibson assembly into FseI-digested pSN150-Int1 to produce pSN150-Int2 (*Supplementary Methods Figure 5C*). Lastly, an sgRNA gblock (IDT) consisting a [5'-cMyc tag]-[T7 promoter]-(I-ppoI/AflII restriction site)-[sgRNA scaffold]-[T7 terminator]- [3'-HA tag] was installed by Gibson assembly into BsiWI-digested pSN150-Int2 to create pSN150 (*Supplementary Methods Figure 5D*).

pSN053 and pSN054 construction

The pSN053-gblock was cloned by Gibson assembly into FseI+SacII-digested pSwing to yield pSN053-Int1 (*Supplementary Methods Figure 6A,B*). A fragment containing the *hsp86* 5'*UTR*-TetR-DOZI$_{2A}$-RLuc$_{2A}$BSD-*hrp2* 3'*UTR* transcription unit was released from pSN1847 by NotI+SacI digestion, and inserted by Gibson assembly into ApaI-digested pSN053-Int1 to yield pSN053-Int2 (*Supplementary Methods Figure 6C*). Lastly, a fragment containing c-Myc-HA-FLAG-10x TetR aptamer-*hsp86* 3'UTR was released from pSN154 by DraIII+SalI digestion and installed by Gibson assembly into SacII-digested pSN053-Int2 to yield pSN053 (*Supplementary Methods Figure 6D*).

To obtain pSN054, I-SceI-digested pSN053 was Gibson assembled with pSN054-conversion gblock to yield pSN054. Thus, pSN054 differs from pSN053 in that the I-SceI site is immediately downstream of SEQ3 and SEQX is deleted (*Supplementary Methods Figure 6E*).

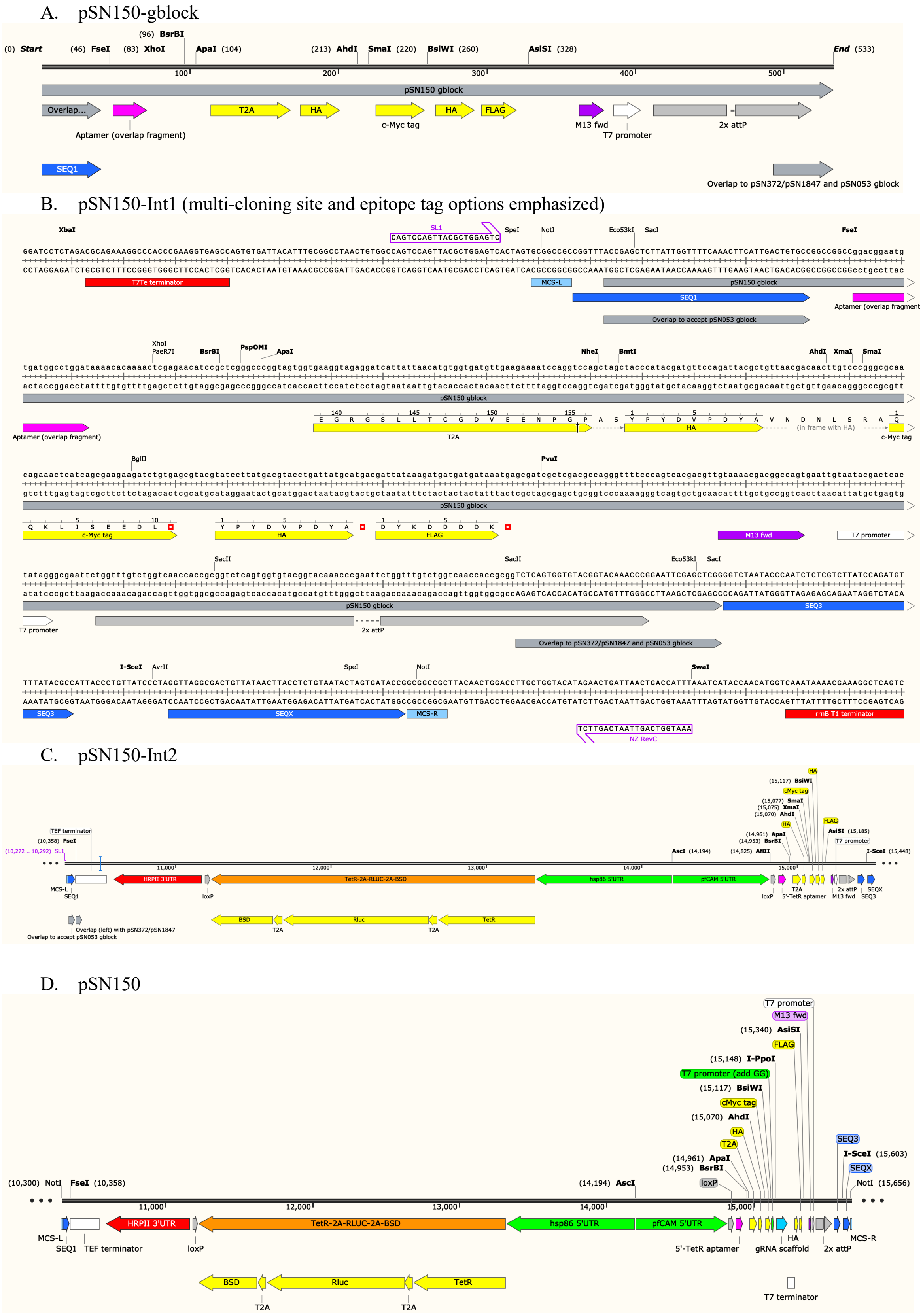

Supplementary Methods Figure 5. **Overview of pSN150 construction.** Key features are shown for (A) pSN150 gblock, (B) pSN150-Int1, (C) pSN150-Int2 and (D) pSN150.

A. pSN053-gblock

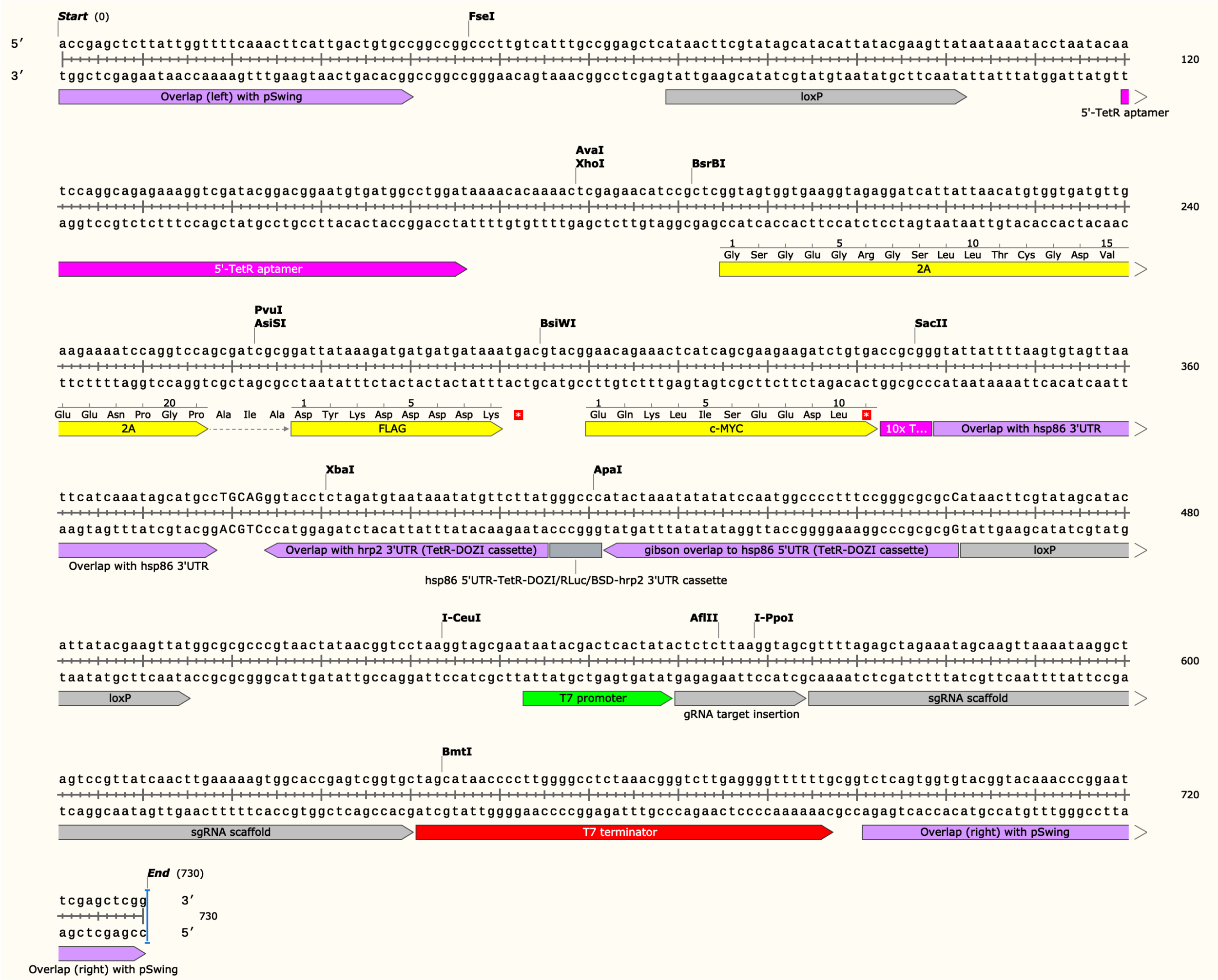

B. pSN053-Int1

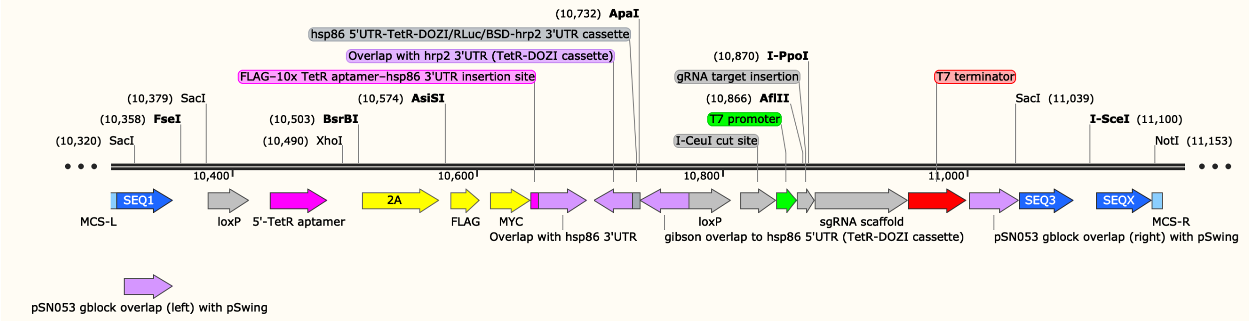

C. pSN053-Int2

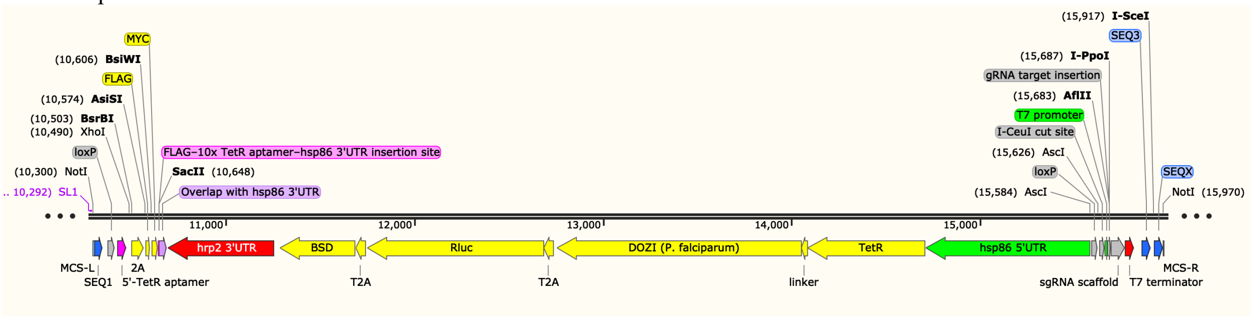

D. pSN053

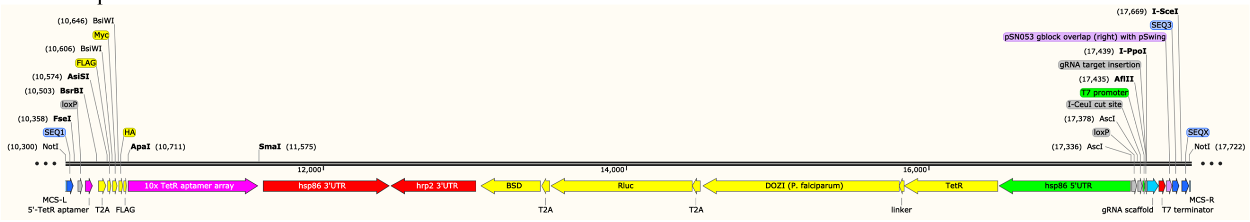

E. Conversion of pSN053 into pSN054

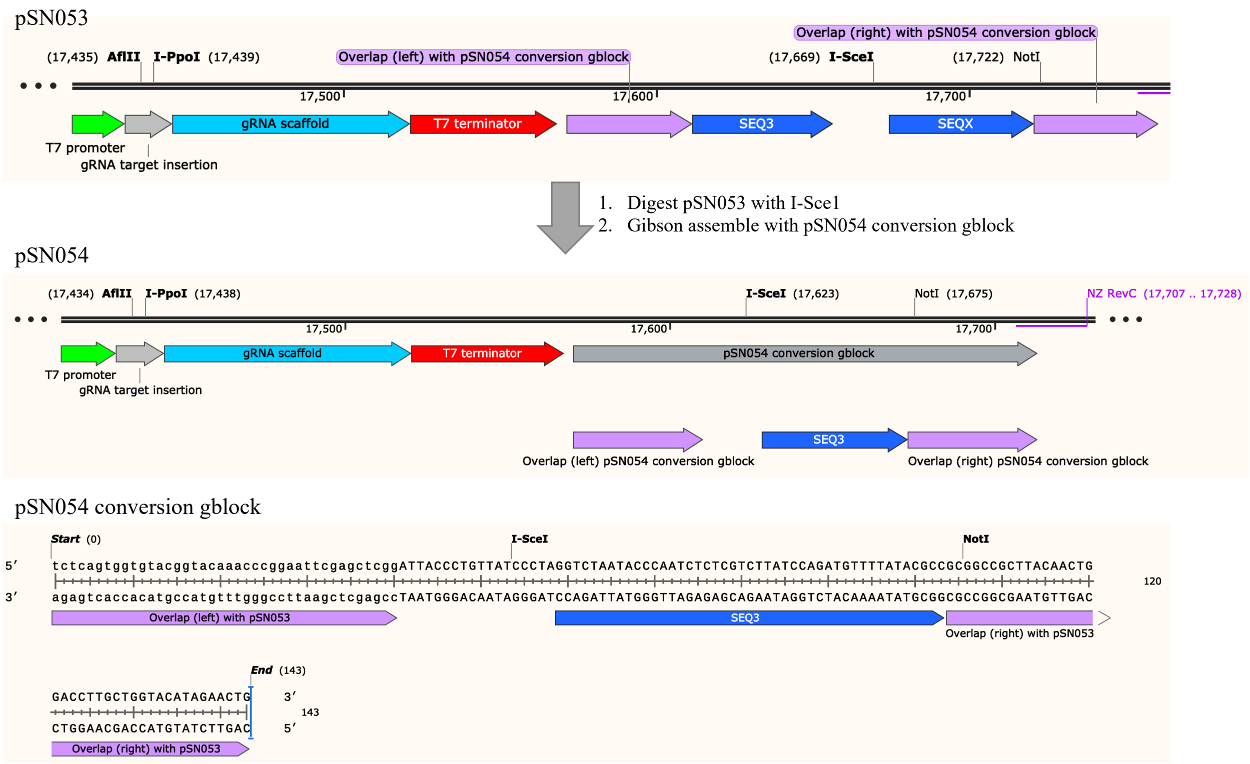

Supplementary Methods Figure 6. Overview of pSN053/4 construction. Key features are shown for (A) pSN053 gblock, (B) pSN053-Int-1, (C) pSN053-Int2, (D) pSN053 and (E) pSN053 conversion to pSN054.

Section 2. Detailed methods for configuring each linear base vector (pSN154, pSN150, pSN053/pSN054) to achieve diverse locus modification outcomes in *P. falciparum*.

A. Assembling complementation/over-expression vectors for episomal maintenance in pSN154 *(Estimated time: 7 days)*

Day 1

1. Digest 200 ng pSN154 plasmid with the appropriate enzyme depending on one's choice of epitope tag/tags (see table 1) in 10 µL at 37 ºC for 90 min, followed by heat inactivation of enzymes at the appropriate temperature.

**Restriction enzymes**	**Tag on N-terminus**	**Tag on C-terminus**
AsiSI only	-	HA+T2A
AsiSI+BsrBI	-	T2A
AsiSI+DraIII; BsrBI+DraIII; DraIII	-	cMyc, HA or FLAG
AsiSI+BsiWI; BsrBI+BsiWI; DraIII+BsiWI	-	HA or FLAG
BsrBI	HA	T2A sequence or none if desired

2. PCR the full-length gene of interest from genomic DNA (gDNA) or complementary DNA (cDNA) of the *P. falciparum* strain being studied with a high-fidelity polymerase. Gel purify the PCR product. Resuspend to 20 ng/µL in water.
3. Mix 20 ng each of PCR product and digested vector with 3 µL 2x Gibson Master Mix and incubate reaction at 50 ºC for 1 h.
4. Transform Big Easy TSA cells with 1 µL Gibson reaction mixture Transformation conditions: 1 mm cuvette, 10 µF, 600 Ohms, 1800 V.
5. Plate on LB-agar with chloramphenicol (34 µg/mL) and incubate overnight at 30 ºC. [**Note**: Big Easy TSA cells are ampicillin resistant.]

Day 2

Pick 8 colonies and grow overnight at 30 ºC in liquid LB media containing chloramphenicol (34 µg/mL) and 0.2% w/v arabinose.

Day 3

1. Evaluate insertion of the gene of interest with an AflII and ApaI double digest.
2. Confirm correctly digesting plasmids by sequencing the inserted gene and its flanking sequences.

Day 4

1. Digest 200 ng of the linear plasmid with NotI and I-SceI in a 10 µL volume at 37 ºC for 90 min followed by heat inactivation of enzymes at 80 ºC.

2. Digest 200 ng of pBigBOB with PacI and heat inactivate enzyme at 65 °C for 20 min.
3. Digest 200 ng of pAdapter with XhoI and XbaI at 37 ºC for 3 h and gel purify the plasmid backbone. Resuspend to 20 ng/µL. **NOTE**: pAdapter confers kanamycin resistance and XhoI cannot be heat inactivated.

4. Incubate 20 ng of each of the digested plasmid containing gene of interest, pBigBOB and pAdapter in a Gibson reaction at 50 ºC for 1 h.
5. Transform BAC-Optimized Replicator v2.0 Electrocompetent cells with 1 µL Gibson reaction mixture. Transformation conditions: 1 mm cuvette, 10 µF, 600 Ohms and 1800 V.
6. Plate on LB-agar with chloramphenicol (34 µg/mL) and kanamycin (50 µg/mL) and incubate overnight at 30 ºC.

Day 5

Pick 8 colonies and grow overnight at 30 ºC in liquid LB media containing chloramphenicol (34 µg/mL) and kanamycin (50 µg/mL).

Day 6

1. Pick colonies and mini-prep plasmid DNA.
2. Digest with AvrII, PvuI and NotI+XhoI to confirm that the relevant cassettes are present.

B. Assembling vectors to engineer promoter and *5′-UTR* regions of *P. falciparum* loci using pSN150 *(Estimated time: 10 days)*

Day 1: Installing recoded coding sequence (CDS) and right homologous regions (RHR).

1. Digest 200 ng pSN150 plasmid with the desired enzymes below in 10 µL at 37 ºC for 90 min, followed by heat inactivation of restriction enzyme(s) at 80 ºC for 20 min.

**Restriction enzymes**	**Tag on N-terminus**
BsrBI+AhdI/BsiWI	None
AhdI; AhdI+BsiWI	HA
BsiWI	HA or cMyc

2. PCR the RHR from gDNA using a high-fidelity polymerase and gel purify the product. Resuspend to 20 ng/µL.
3. Resuspend synthetic DNA corresponding to recoded CDS region in water to 20 ng/µL.
4. Mix 20 ng each of the RHR, gblock and digested vector with an equal volume 2x Gibson Assembly master mix. Incubate at 50 ºC for 1 h.
5. Transform BigEasy-TSA Electrocompetent Cells with 1 µL Gibson reaction mixture. Transformation conditions: 1 mm cuvette, 10 µF, 600 Ohms, 1800 V.
6. Plate on LB-agar with chloramphenicol (34 µg/mL) and incubate overnight at 30 ºC.

Day 2

Pick 8 colonies and grow overnight at 30 ºC in liquid LB media containing chloramphenicol (34 µg/mL) and 0.2% w/v arabinose.

Day 3

1. Mini-prep plasmid DNA and test for insertion of both RHR and recoded DNA segment by restriction digestion with AflII and BsiWI.
2. Confirm correctly digesting plasmids by DNA sequencing and select the pSN150-RHR intermediate for the next step.

Day 4: Installation of the left homologous region (LHR)

1. Digest 200 ng pSN150-RHR plasmid with FseI in 10 µL at 37 ºC for 90 min followed by heat inactivation of enzyme at 80 ºC for 20 min.
2. PCR the LHR from gDNA using a high-fidelity polymerase and gel purify the product. Resuspend to 20 ng/µL.
3. Mix 20 ng LHR and digested vector with 3 µL 2x Gibson Assembly master mix, and incubate at 50 ºC for 1 h.
4. Transform Big Easy TSA cells with 1 µL Gibson reaction mixture.
5. Plate on LB-agar with chloramphenicol (34 µg/mL) and incubate overnight at 30 ºC.

Day 5

Pick 8 colonies and grow overnight at 30 ºC in liquid LB media containing chloramphenicol (34 µg/mL) and 0.2% w/v arabinose.

Day 6

1. Mini-prep plasmid DNA and analyze colonies for LHR insertion NotI+AscI digest and RHR/recoded region retention AflII+BsiWI digest.
2. Confirm correctly digesting plasmids by sequencing the LHR and select pSN150-LHR-RHR intermediate for the next step.

Day 7: Installation of sgRNA into pSN150-LHR-RHR intermediate.

1. Digest 200 ng of the vector with I-ppoI or AflII.
2. Obtain sgRNA-encoding DNA fragment using complementary primers in a Klenow reaction (see protocol below) or purchase as synthetic DNA fragment. (*Optional*: The Klenow fragment can be agarose gel purified, if needed).
3. Mix 20 ng each sgRNA-encoding DNA and digested pSN150-LHR-RHR vector with 3 µL 2x Gibson Assembly master mix and incubate at 50 ºC for 1 h.
4. Transform BigEasy-TSA Electrocompetent Cells with 1 µL Gibson reaction mixture. Transformation conditions: 1 mm cuvette, 10 µF, 600 Ohms, 1800 V.
5. Plate on LB-agar with chloramphenicol (34 µg/mL) and incubate overnight at 30 ºC.

Day 8

Pick 8 colonies and grow overnight at 30 ºC in liquid LB media containing chloramphenicol (34 µg/mL) and 0.2% w/v arabinose.

Day 9

1. Mini-prep plasmid DNA and analyze colonies for sgRNA-encoding DNA insertion by AflII (or I-ppoI) digest.
2. Sequence sgRNA insert using the T7 promoter or other suitable primer.
3. Confirm retention of inserted LHR and RHR regions using (NotI+AscI) and AflII+BsiWI restriction digests, respectively, in selected final colonies.

Day 10

Maxi-prep plasmid for *P. falciparum* transfection. Plasmids are quite stable during this step. However, as prudent practice, we recommend performing restriction digests as in the previous step to verify the absence of gross deletions/rearrangements.

C. Assembling donor vectors to achieve gene deletions in *P. falciparum* using pSN150
(Estimated time: 10 days)

Day 1: Installation of left homologous region (LHR)

1. Digest 200 ng pSN150 plasmid with FseI in 10 µL volume at 37 ºC for 90 min, followed by heat inactivation of enzyme at 80 ºC for 20 min.
2. PCR the LHR from gDNA using a high-fidelity polymerase and gel purify the product. Resuspend to 20 ng/µL.
3. Mix 20 ng each of LHR and digested vector with 3 µL 2x Gibson Assembly master mix, and incubate at 50 ºC for 1 h.
4. Transform BigEasy-TSA Electrocompetent Cells with 1 µL Gibson reaction mixture. Transformation conditions: 1 mm cuvette, 10 µF, 600 Ohms, 1800 V.
5. Plate on LB-agar with chloramphenicol (34 µg/mL) and incubate overnight at 30 ºC.

Day 2

Pick 8 colonies and grow overnight at 30 ºC in liquid LB media containing chloramphenicol (34 µg/mL) and 0.2% w/v arabinose.

Day 3

1. Mini-prep plasmid DNA and test for LHR insertion by restriction digestion with FseI+XbaI (or XbaI alone). [**Note**. Even though the XbaI site is not unique in this plasmid, digesting with this enzyme will produce a pattern unambiguously identifying LHR insertion.]
2. Confirm correctly digesting plasmids by sequencing the LHR, and select the pSN150-LHR intermediate for the next step.

Day 4: Installation of right homologous region (RHR)

1. Digest 200 ng pSN150-LHR plasmid with AscI+XmaI in a 10 µL at 37 ºC for 90 min, followed by heat inactivation of enzymes at 80 ºC for 20 min.
2. PCR the RHR from gDNA using a high-fidelity polymerase and gel purify the product. Resuspend to 20 ng/µL.
3. Mix 20 ng each of the RHR fragment and digested vector pSN150-LHR with 3 µL 2x Gibson Assembly master mix at 50 ºC for 1 h.
4. Transform BigEasy-TSA Electrocompetent Cells with 1 µL Gibson reaction mixture. Transformation conditions: 1 mm cuvette, 10 µF, 600 Ohms, 1800 V.
5. Plate on LB-agar with chloramphenicol (34 µg/mL) and incubate overnight at 30 ºC.

Day 5

Pick 8 colonies and grow overnight at 30 ºC in liquid LB media containing chloramphenicol (34 µg/mL) and 0.2% w/v arabinose.

Day 6

1. Mini-prep plasmid DNA and analyze for LHR and RHR insertion FseI+XbaI and AscI+BsiWI digests, respectively.
2. Confirm correctly digesting plasmids by sequencing the RHR, and select pSN150-LHR-RHR intermediate for the next step.

Day 7: Installation of DNA encoding the sgRNA

1. Digest 200 ng pSN150-LHR-RHR plasmid with I-ppoI (Promega) in 10 µL at 37 ºC for 90 min, followed by heat inactivation of enzyme at 80 ºC for 20 min.
2. Obtain sgRNA-encoding DNA fragment using complementary primers in a Klenow reaction (see protocol below) or purchase as synthetic DNA fragment. (*Optional*: The Klenow fragment can be agarose gel purified, if desired.)
3. Mix 20 ng each of sgRNA-encoding DNA and digested pSN150-LHR-RHR vector with 3 µL 2x Gibson Assembly master mix and incubate at 50 ºC for 1 h.
4. Transform BigEasy-TSA Electrocompetent Cells with 1 µL of the Gibson reaction mixture. Transformation conditions: 1 mm cuvette, 10 µF, 600 Ohms, 1800 V.
5. Plate on LB-agar with chloramphenicol (34 µg/mL) and incubate overnight at 30 ºC.

Day 8

Pick 8 colonies and grow overnight at 30 ºC in liquid LB media containing chloramphenicol (34 µg/mL) and 0.2% w/v arabinose.

Day 9

1. Mini-prep plasmid DNA and analyze for sgRNA-encoding DNA insertion by I-ppoI digest.
2. Sequence digest-positive plasmids to confirm the correct sgRNA sequence using the T7 promoter or AF443 primer.
3. Confirm retention of inserted LHR and RHR regions using FseI+XbaI and AscI+BsiWI restriction digests, respectively, in selected final plasmids.

Day 10

Maxi-prep final plasmid to obtain DNA for *P. falciparum* transfections. Plasmids are quite stable during this step. However, as prudent practice, we recommend performing restriction digests as in the previous step to verify the absence of gross deletions/rearrangements.

D. Assembling donor vectors to engineer the *3′-UTR* of *P. falciparum* loci using pSN053/054
(Estimated time: 10 days)

Day 1: Installation of right homologous region (RHR)

1. Digest 200 ng pSN053/054 plasmid with I-SceI in a 10 µL at 37 ºC for 90 min, followed by heat inactivation of enzyme at 80 ºC for 20 min.
2. PCR the RHR from gDNA using a high-fidelity polymerase and gel purify the product. Resuspend product to 20 ng/µL.
3. Mix 20 ng each of the RHR and digested vector in 3 µL 2x Gibson Assembly master mix and incubate at 50 ºC for 1 h.
4. Transform BigEasy-TSA Electrocompetent Cells with 1 µL Gibson reaction mixture. Transformation conditions: 1 mm cuvette, 10 µF, 600 Ohms, 1800 V.
5. Plate on LB-agar with chloramphenicol (34 µg/mL) and incubate overnight at 30 ºC.

Day 2

Pick 8 colonies and grow overnight at 30 ºC in liquid LB media containing chloramphenicol (34 µg/mL) and 0.2% w/v arabinose.

Day 3

1. Mini-prep plasmid DNA and test for RHR insertion by AflII digestion to look for a shift in size of the smaller part of the plasmid (downstream of AflII).
2. Sequence inserted RHR region for correctly digesting plasmids, and select a pSN053/54-RHR intermediate plasmid for the next step.

Day 4: Installation of left homologous region (LHR)

1. Digest 200 ng pSN053/53-RHR with FseI+BsrBI, FseI+AsiSI or FseI+BsiWI (to include either a T2A sequence, FLAG or Myc/HA epitope tag, respectively) in 10 µL at 37 ºC for 90 min, followed by heat inactivation of enzymes at the appropriate temperatures for this enzyme combination.
2. PCR amplify the LHR from gDNA using a high-fidelity polymerase and gel purify. Resuspend to 20 ng/µL.
3. Mix 20 ng each of LHR and digested vector in 3 µL 2x Gibson Assembly master mix,and incubate at 50 ºC for 1 h.
4. Transform BigEasy-TSA Electrocompetent Cells with 1 µL of Gibson reaction mixture. Transformation conditions: 1 mm cuvette, 10 µF, 600 Ohms, 1800 V.
5. Plate on LB-agar with chloramphenicol (34 µg/mL) and incubate overnight at 30 ºC.

Day 5

Pick 8 colonies and grow overnight at 30 ºC in liquid LB media containing chloramphenicol (34 µg/mL) and 0.2% w/v arabinose.

Day 6

1. Mini-prep plasmid DNA and analyze for LHR insertion (NotI+BsiWI digest) and RHR retention (AflII digest- look for a shift in size downstream of Aflll).
2. Confirm correctly digesting plasmids by sequencing the LHR, and select pSN150-LHR-RHR intermediate for the next step.

Day 7: Installation of DNA encoding the sgRNA

1. Digest 200 ng pSN053/054-LHR-RHR plasmid with AflII or I-ppoI in 10 µL at 37 ºC for 90 min, followed by heat inactivation of enzyme at 80 ºC for 20 min.
2. Obtain sgRNA-encoding DNA fragment using complementary primers in a Klenow reaction (see protocol below) or purchase as synthetic DNA fragment. *Optional*: The Klenow fragment can be agarose gel purified, if desired.
3. Mix 20 ng each sgRNA-encoding DNA and digested pSN150-LHR-RHR vector with 3 µL 2x Gibson Assembly master mix and incubate at 50 ºC for 1 h.
4. Transform BigEasy-TSA Electrocompetent Cells using 1 µL Gibson reaction mixture. Transformation conditions: 1 mm cuvette, 10 µF, 600 Ohms, 1800 V.
5. Plate on LB-agar with chloramphenicol (34 µg/mL) and incubate overnight at 30 ºC.

Day 8

Pick 8 colonies and grow overnight at 30 ºC in liquid LB media containing chloramphenicol (34 µg/mL) and 0.2% w/v arabinose.

Day 9

1. Mini-prep plasmid DNA and analyze for sgRNA-encoding DNA insertion by AflII (or I-ppoI) digest.
2. Sequence digest-positive plasmids to verify the sgRNA sequence using T7 promoter or other user-designed primer.
3. Confirm retention of inserted LHR and RHR regions using NotI+BsiWI and AscI+I-SceI restriction digests, respectively, in selected final plasmids.

Day 10

Maxi-prep final plasmid to obtain DNA for *P. falciparum* transfections. Plasmids are quite stable during this step. However, as prudent practice, we recommend performing restriction digests as in the previous step to verify the absence of gross deletions/rearrangements.

E. Assembling donor vectors to achieve dual TetR aptamer-mediated target gene regulation at their native loci using pSN053/054 *(Estimated time: 13 days)*

Day 1: Installation of right homologous region (RHR)

1. Digest 200 ng pSN053/054 plasmid with I-SceI in 10 µL at 37 ºC for 90 min, followed by heat inactivation of enzyme at 80 ºC for 20 min.
2. PCR the RHR from gDNA using a high-fidelity polymerase and gel purify. Resuspend to 20 ng/µL.
3. Mix 20 ng each of RHR and digested vector with 3 µL 2x Gibson Assembly master mix and incubate at 50 ºC for 1 h.
4. Transform BigEasy-TSA Electrocompetent Cells with 1 µL Gibson reaction mixture. Transformation conditions: 1 mm cuvette, 10 µF, 600 Ohms, 1800 V.
5. Plate on LB-agar with chloramphenicol (34 µg/mL) and incubate overnight at 30 ºC.

Day 2

Pick 8 colonies and grow overnight at 30 ºC in liquid LB media containing chloramphenicol (34 µg/mL) and 0.2% w/v arabinose.

Day 3

1. Mini-prep plasmid DNA and test for RHR insertion by AflII restriction digestion.
2. Sequence inserted RHR region of correctly digesting plasmids, and select a pSN053/54-RHR intermediate plasmid for the next step.

Day 4: Installation of left homologous region (LHR)

1. Digest 200 ng pSN053/054-RHR with FseI in 10 µL at 37 ºC for 90 min, followed by heat inactivation of enzyme at 80 ºC for 20 min.
2. PCR amplify LHR from gDNA using a high-fidelity polymerase and gel purify. Resuspend to 20 ng/µL.
3. Mix 20 ng each of LHR and digested vector in 3 µL 2x Gibson Assembly master mix, and incubate at 50 ºC for 1 h.
4. Transform BigEasy-TSA Electrocompetent Cells with 1 µL Gibson reaction mixture. Transformation conditions: 1 mm cuvette, 10 µF, 600 Ohms, 1800 V.
5. Plate on LB-agar with chloramphenicol (34 µg/mL) and incubate overnight at 30 ºC.

Day 5

Pick 8 colonies and grow overnight at 30 ºC in liquid LB media containing chloramphenicol (34 µg/mL) and 0.2% w/v arabinose.

Day 6

1. Mini-prep plasmid DNA and analyze colonies for LHR insertion NotI+FseI digest and RHR retention AflII digest.
2. Confirm LHR sequence for correctly digesting plasmids, and select pSN150-LHR-RHR intermediate for the next step.

Day 7: Installation of recoded DNA encoding CDS for gene of interest

1. Digest 200 ng pSN053/054-LHR-RHR vector with BsrBI/AsiSI/BsiWI in 10 µL at 37 ºC for 90 min, followed by heat inactivation of enzyme(s) at the appropriate temperature for 20 min. See below the epitope tags that can be used based on the restriction enzyme used.

**Restriction enzymes**	**Tag on N-terminus**	**Tag on C-terminus**
BsrBI	none	FLAG, c-Myc or HA
AsiSI	none	FLAG, c-Myc or HA
BsiWI	± FLAG	c-Myc or HA

2. Resuspend the synthetic gene to 20 ng/µL in water. Note: ensure the synthetic gene has Gibson homology to the left and right Gibson regions.
3. Mix 20 ng each of synthetic gene DNA and digested vector with 3 µL 2x Gibson Assembly master mix and incubate at 50 ºC for 1 h.
4. Transform BigEasy-TSA Electrocompetent Cells with 1 µL of Gibson reaction mixture. Transformation conditions: 1 mm cuvette, 10 µF, 600 Ohms, 1800 V.
5. Plate on LB-agar with chloramphenicol (34 µg/mL) and incubate overnight at 30 ºC.

Day 8

Pick 8 colonies and grow overnight at 30 ºC in liquid LB media containing chloramphenicol (34 µg/mL) and 0.2% w/v arabinose.

Day 9

1. Mini-prep plasmid DNA and analyze colonies for insertion of recodonized CDS by BsiWI and NotI digestion. Use the pSN053/054-LHR-RHR plasmid as a control to detect increase in size of diagnostic fragment containing the inserted CDS.
2. Sequence positive clones to verify correct CDS sequence.
3. Confirm retention of LHR and RHR regions using NotI+BsrBI and AflII restriction digests, respectively.
4. Select pSN053/054-LHR-rCDS-RHR intermediate plasmid for the next step.

Day 10: Installation of DNA encoding the sgRNA

1. Digest 200 ng pSN053/054-LHR-rCDS-RHR plasmid with AflII or I-ppoI in 10 µL at 37 ºC for 90 min, followed by heat inactivation of enzyme at 80 ºC for 20 min.
2. Obtain sgRNA-encoding DNA fragment using complementary primers in a Klenow reaction (see protocol below) or purchase as synthetic DNA fragment. *Optional*: The Klenow fragment can be agarose gel purified, if desired.
3. Mix 20 ng each sgRNA-encoding DNA and digested pSN053/054-LHR-rCDS-RHR vector with 3 µL 2x Gibson Assembly master mix and incubate at 50 ºC for 1 h.
4. Transform BigEasy-TSA Electrocompetent Cells with 1 µL of the Gibson reaction mixture. Transformation conditions: 1 mm cuvette, 10 µF, 600 Ohms, 1800 V.
5. Plate on LB-agar with chloramphenicol (34 µg/mL) and incubate overnight at 30 ºC.

Day 11

Pick 8 colonies and grow overnight at 30 ºC in liquid LB media containing chloramphenicol (34 µg/mL) and 0.2% w/v arabinose.

Day 12

1. Mini-prep plasmid DNA and analyze colonies for sgRNA-encoding DNA insertion by AflII (or I-ppoI) digestion.
2. Sequence digest-positive clones to verify correct sgRNA identity using T7 promoter or other user-designed primer.
3. Confirm retention of inserted LHR, rCDS and RHR regions using NotI+BsrBI, BsrBI+XmaI and I-CeuI restriction digests, respectively, in the selected final pSN053-LHR-rCDS-RHR-sgRNA/pSN054-LHR-rCDS-sgRNA-RHR plasmids.

Day 13

Maxi-prep final plasmids to obtain DNA for *P. falciparum* transfections. Plasmids are quite stable during this step. However, as prudent practice, we recommend performing restriction digests as in the previous step to verify the absence of gross deletions/rearrangements.

F. Rescuing linear plasmids into BACS *(Estimated time: 5 days)*

Day 1

1. Digest 200 ng linear vector construct with I-SceI+NotI at 37 °C for 90 min and inactivate restriction enzymes for 20 min at 80 °C.
2. Overnight digest 200 ng pBigBOB and pAdapter with PacI and XhoI+XbaI, respectively, in 10 µL reactions at 37 °C, followed by heat inactivation of enzymes at 80 °C for 20 min. Perform all reactions in 10ul volumes. **Note**: Since pAdapter and pBigBoB are used in all rescue reactions, larger scale preparations can be performed. Digested vectors stored at -20 °C for future experiments.

Day 2

1. Combine 1 µL each of digested linear plasmid, pAdapter and pBigBoB with 3 µL 2x Gibson Assembly Master Mix and incubate at 50 °C for 1 h.
2. Transform BAC-Optimized Replicator v2.0 Electrocompetent cells with 1 µL Gibson reaction.
3. Plate on LB-agar with chloramphenicol (34 µg/mL) and kanamycin (50 µg/mL) and incubate overnight at 30 °C.

Day 3

Pick 8 colonies and grow overnight at 30 ºC in liquid LB media containing chloramphenicol (34 µg/mL) and kanamycin (50 µg/mL)

Day 4

1. Mini-prep BAC DNA and test for insertion of the fragment released from the linear vector during I-SceI+NotI digestion (Day1, Step 1).
2. Confirm proper overall BAC topology using AvrII, PvuI and NotI/XhoI digests.

Day 5

Maxi-prep final BAC to obtain DNA for *P. falciparum* transfections. These are quite stable during this step. However, as prudent practice, we recommend repeating restriction digests as in the previous step to verify absence of gross deletions/rearrangements.

Supplementary Figures

Supplementary Figure 1. Several operations using the pSN vector family for achieving TetR aptamer-regulated conditional regulation of gene expression after genome editing are summarized. (**A**) Expressing a transgene from pSN154 rescued into a BAC that is then either stably maintained as an episome or integrated at a chromosomal *attB* site using the *Bxb1* integrase. (**B**) Using pSN150 and CRISPR-Cas9 genome editing to swap a native promoter for a synthetic promoter regulated by a single TetR aptamer in the 5′-*UTR* to conditionally regulate expression of an endogenous gene from its native chromosomal locus. (**C**) Using pSN053/54 and CRISPR/Cas9 genome editing to place a gene transcribed by its native promoter under dual TetR aptamer (5′- and 3′-*UTR* located) conditional regulation by TetR-DOZI. (**D**) Using pSN053/54 and CRISPR/Cas9 genome editing to place a gene transcribed by its native promoter under conditional regulation by TetR-DOZI via 3′-*UTR* located TetR aptamers.

Supplementary Figure 2. Complete Sanger sequencing traces of the (**A**) 5′- and (**B**) 3′-junction integration PCR products from the *eba175* knockout parasite line.

Supplementary Figure 3. PCR analysis of clones derived from transfections in which pSN150-rescued BACs co-transfected with the pCRISPR plasmid are used to edit the GSK3, HT and TrxR loci.

Supplementary Figure 4. Uncropped PCR gels and Western blot images for pSN150 modification constructs. (**A-E**) Uncropped PCR agarose gel images corresponding to those

presented in Figure 5C. (**F-I**) Uncropped blots corresponding to those presented in Figure 5D but merged with the molecular weight marker detected using colorimetric imaging. The blots were initially probed with mouse anti-HA antibody (top panels), re-blocked, and probed with rabbit anti-GAPDH (bottom panels). Short and long membrane exposure conditions were carried out for optimal detection and presentation of CK and CRT signals.

Supplementary Figure 5. Growth of parasites in which the HT locus has been engineered to achieve regulated expression via a non-native, synthetic promoter/*5′-UTR* regulated by a single TetR aptamer within the transcript's *5′-UTR* (pSN150) or its native promoter and a 10x aptamer array positioned within the *3′-UTR* of the transcript (pSN054). The relative growth of ring stage parasites inoculated at varying initial parasitemia (0.1%, 0.5% and 1.5%) in media containing a range of glucose (0.2, 0.8, 1.4 and 2.0 mg/mL) and aTc (0, 1, 3 and 50 nM) concentrations was determined after 72 h. Parasite growth was determined by monitoring *Renilla* luciferase levels, with the values obtained at 2 mg/mL glucose and 50 nM aTc condition representing maximal (100%) growth. Data are the mean ± s.d. from one of two independent experiments.

Supplementary Figure 6. Uncropped PCR gels and Western blot images for pSN054 modification constructs. (**A-C**) Uncropped PCR agarose gel images corresponding to those presented in Figure 7B. (**D-E**) Uncropped blots corresponding to those presented in Figure 7C, but merged with the molecular weight marker detected using colorimetric imaging. The blots were initially probed with mouse anti-HA antibody (top panels), re-blocked, and probed with rabbit anti-GAPDH (bottom panels).

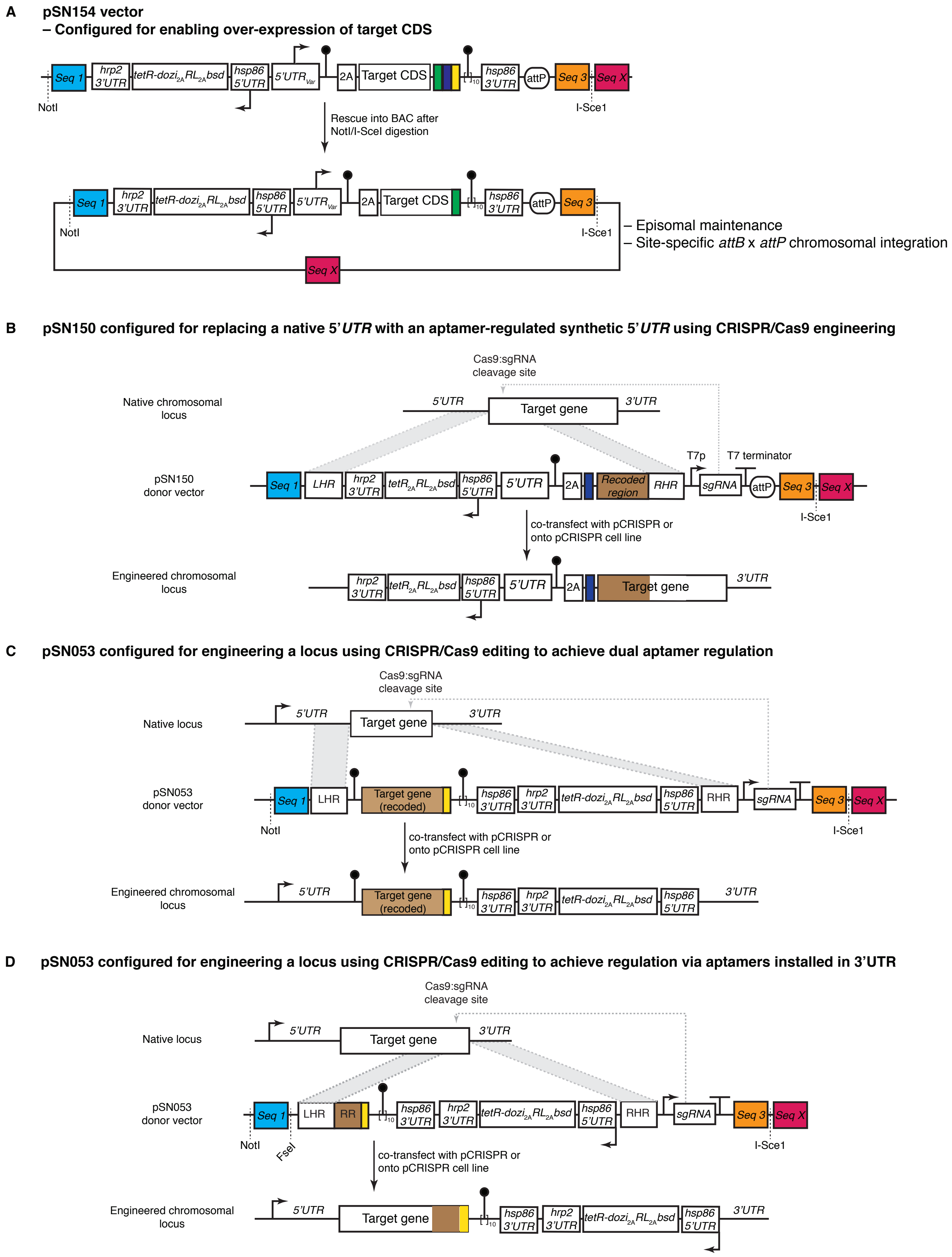

Supplementary Figure 1

Supplementary Figure 2A

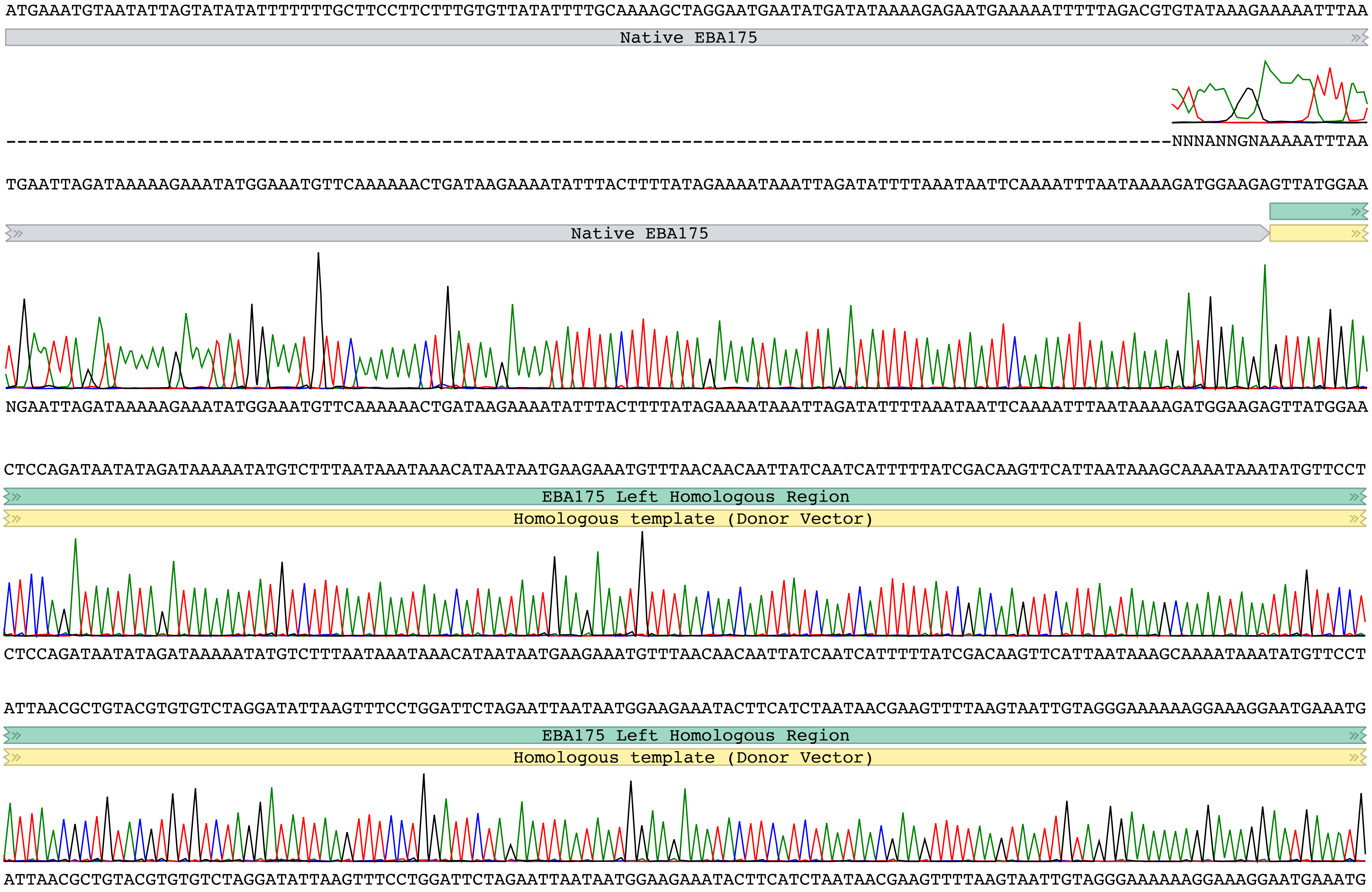

Supplementary Figure 2B

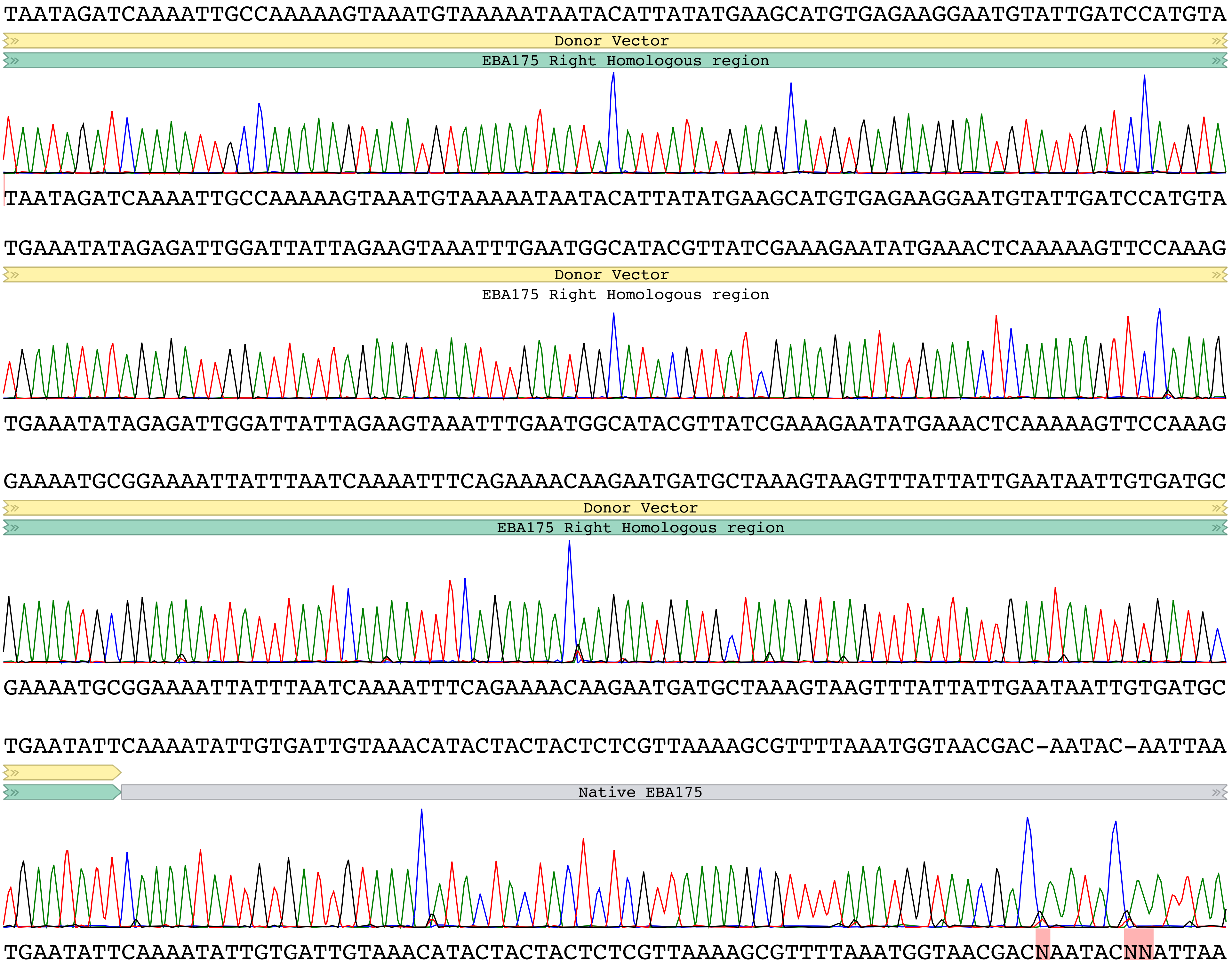

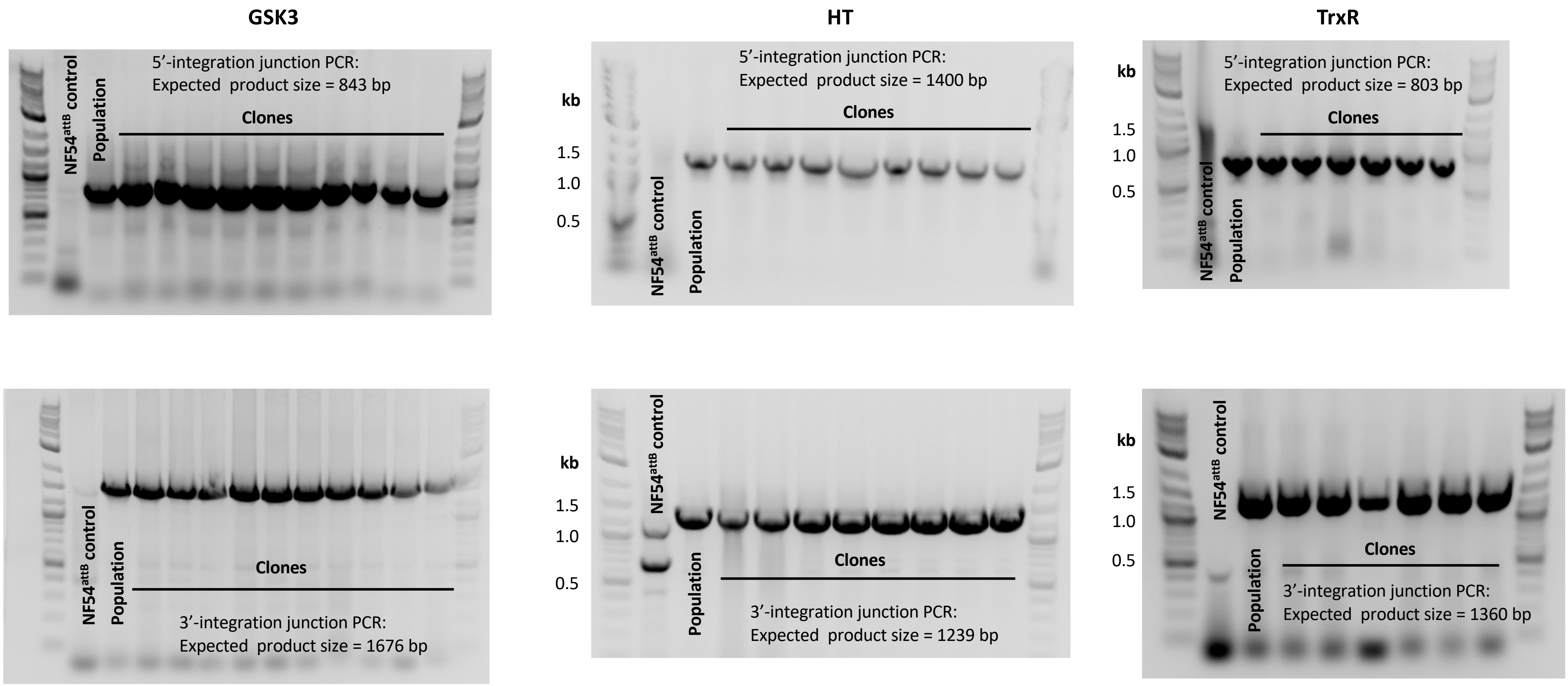

Nasamu *et al*,
Supplementary Figure 3

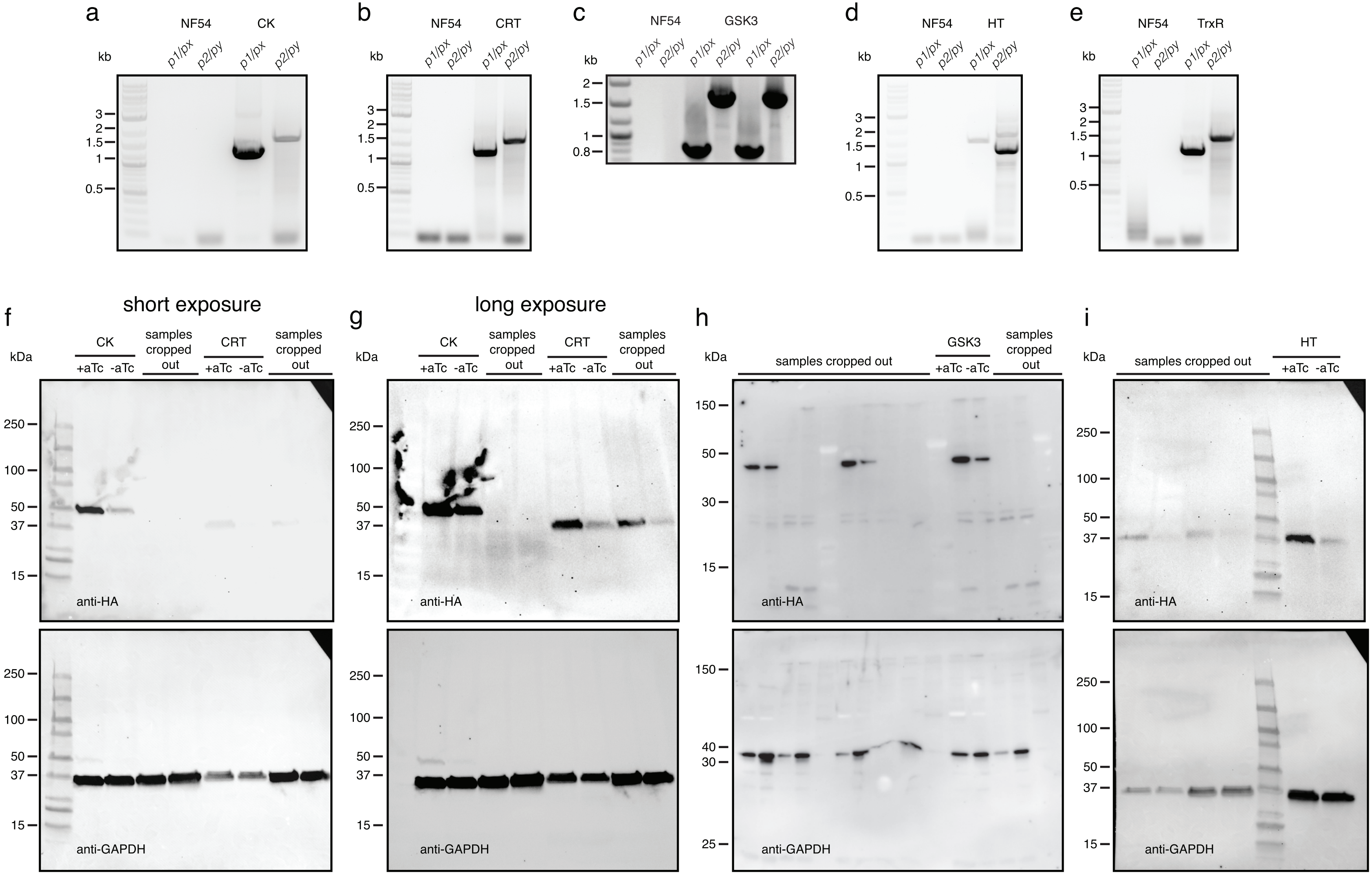

Supplementary Figure 4

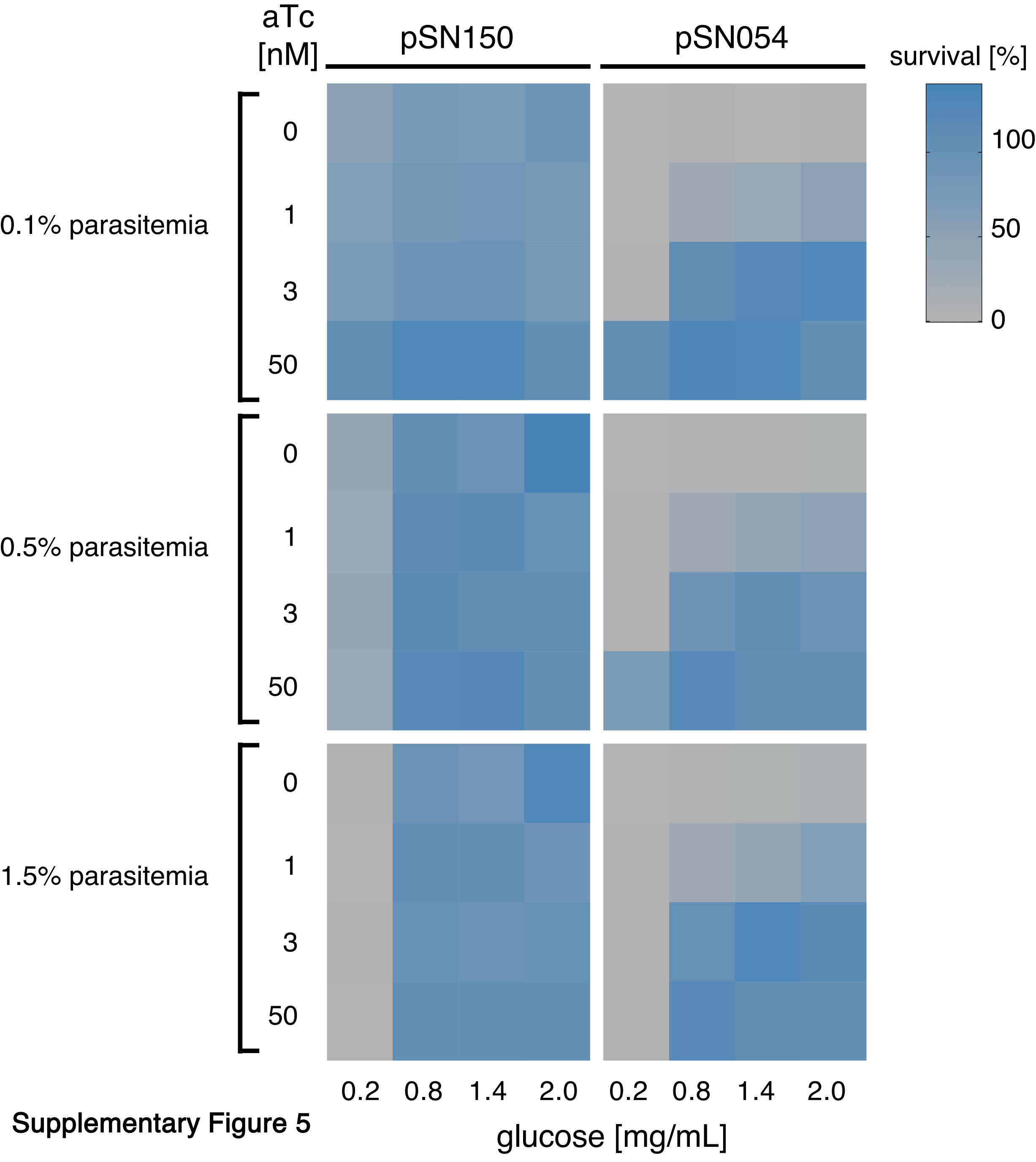

Supplementary Figure 5

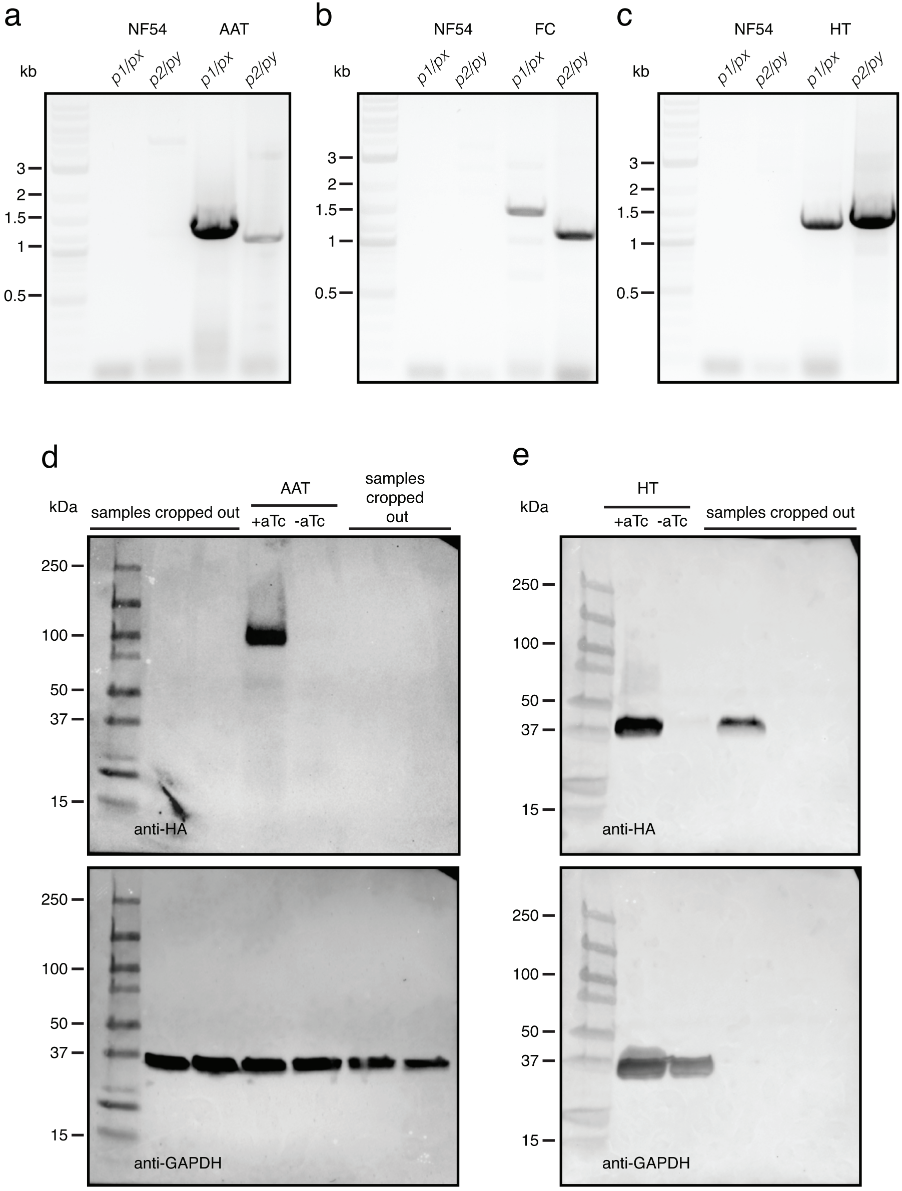

Supplementary Figure 6

Supplementary Table 1. Construction of circular pUF-1 knockout vector for disrupting *eba175* locus.

Description	Nucleotide sequence
eba175_RHR_F	TTCTCAAAAATGAACAATAATTATTCAACTAAGGCAGAAA
eba175_RHR_R	AGTGTAGTTAATTCATCAAATAGCATGCCTGCAGGTCGACAATATTCAGCATCACAATTA
eba175_LHR_F	TATAGAATACTCAAGCTTGGGGGGATCCTCTAGAGTCGACGTTATGGAACTCCAGATAAT
eba175_LHR_R	CCTCTACCTTCACCACTACCCATAGCAAGATGTCCATAAT
eba175_sgRNA target site	GGAAATGATATGGATTTTGG
eba175_LHR integration primer_F	GCTAGGAATGAATATGATATAAAAGAGAATGAAAAATTTTTAGACGTG
eba175_LHR integration primer_R	TGATCTATGGATGTATAACCCTTAGCTTCCATTATGTC
eba175_RHR integration primer_F	TGACAGCCAGTTTAACTACCAAGTTCTTGAAC
eba175_RHR integration primer_R	CCCACACCTTTGTGTTTGTATCAACGG

Supplementary Table 2A. List of oligonucleotides used to construct pSN150 knockdown donor vectors and validate locus-specific integration in edited *P. falciparum* lines.

Description	Nucleotide sequence
CK_RHR_F	TGTGTATTTAAAATGATGGATAGGTGGAGATTAGCTGTAT
CK_RHR_R	TGATGAGTTTCTGTTGCGCCCGGGACAATTAATCGTCATAATCCTTGATAATATTTTTGG
CK_LHR_F	TTTCAAACTTCATTGACTGTGCCGGCCGGCCGTATGTATCACATAAGTTTTGATTTTATC
CK_LHR_R	TCTTGAAAACAAGAATCTTTTTATTGTCCTGTTCTATTAGGTGCTAGATACTAATTTTAC
CK_sgRNA target site	ATGGTGGGCGTATCGAGGAA
CK_LHR integration primer_F	TCAAAGGGAAAACCATAAAATACT
CK_RHR integration primer_R	TTTTTTATATCTCCTTCTTTTTCTACATG
CRT_RHR_F	GGAAATATCCAATCATTTGTTCTTCAATTAAGTATTCC
CRT_RHR_R	CGCTGATGAGTTTCTGTTGCGCCCGGGACAACCCTTGTCATGTTTGAAAAGCATACAGGC
CRT_LHR_F	TTCAAACTTCATTGACTGTGCCGGCCGGCCTTTTTTTTTCCTTTTTTTTACTTTCCCAAG
CRT_LHR_R	TCTTGAAAACAAGAATCTTTTTATTGTCGAATGTATAATAAATAATATGACTGCGTGTAT
CRT_sgRNA target site	GGCTCACGTTTAGGTGG
CRT_LHR integration primer_F	TTCTTATACTTGAACCTTTTTTTTTTTTTT
CRT_RHR integration primer_R	ATATGGTAAATGAACTAAAAAGGGAAAAT
GSK3_RHR_F	CCAATTTTTTCGGGACAGTCAAGTGTGGATCAGCTAGTTA
GSK3_RHR_R	CTGATGAGTTTCTGTTGCGCCCGGGACAAATGCATGGATCTCGTAGTTCATCAAAAAAGG
GSK3_LHR_F	GTTTTCAAACTTCATTGACTGTGCCGGCCGGCCAACCCAAATAGATATACATATAAATAA
GSK3_LHR_R	CTTGAAAACAAGAATCTTTTTATTGTCGTATAATTACAAAATTGACAAAAGGTAATAATC
GSK3_sgRNA target site	GAGTGATCAGAAGGACGAAG
GSK3_LHR integration primer_F	TATACCATCATATTTACAATATCGTGAGTAT
GSK3_RHR integration primer_R	CAAAAGTTGAACAGCTCTGGTAGC
HT_RHR_F	GGCTTATGTTTTTATTTCCTTCTGTCATATCATTAATAGG
HT_RHR_R	GCTGATGAGTTTCTGTTGCGCCCGGGACAATAAATAAGCAACTAAAACTCCTACACATCC
HT_LHR_F	AAACTTCATTGACTGTGCCGGCCGGCCCATTCCTATAGAACATTTTATATAACAAAAATG
HT_LHR_R	TCTTGAAAACAAGAATCTTTTTATTGTCCCACAAACAAATATGAAACGTGAAAATTAATA
HT_sgRNA target site	GGCTCAGTCGAATCAGCCTT
HT_LHR integration primer_F	TTTTTTTTCATGTCACAAATATGGAATAA
HT_RHR integration primer_R	CTGAAAAAACTATGAAAAGAATCGAAG
TrxR_RHR_F	AAGAAATTAGTTACAACTGTACAATCTCACATACGTTCAT
TrxR_RHR_R	CTTCGCTGATGAGTTTCTGTTGCGCCCGGGACAATCAATGTCTCCTTTTCTTCCAATAGC
TrxR_LHR_F	ATTGGTTTTCAAACTTCATTGACTGTGCCGGCCGGCCGTGGGTGGACTTTAAGTTTAGTT
TrxR_LHR_R	GTTCTTGAAAACAAGAATCTTTTTATTGTCGCCAAATGTGTAAAAATAAAAAAAGAGAGA
TrxR_sgRNA target site	AGGAGGAGGTCCAGGTGGAA
TrxR_LHR integration primer_F	GTAAAAATGTGTTATTCTTAACTTTGATATTGAG
TrxR_RHR integration primer_R	TATGCTTTTTCTTCTGAATATCCACAT
pSN150_LHR integration_R	TGAACATAAAGTACAACATTAATATATAGC

pSN150_RHR integration_F	TAAATACCTAATACAATCCAGGCAGA

Legend: LHR, left homology region; RHR, right homology region; F, forward; and R, Reverse.

Supplementary Table 2B. Recoded regions (*T. gondii* codon composition) used during to construct pSN150 knockdown donor vectors.

<u>CK recoded region (bp 1-687)</u>
ATGgagagtaaaatctgcgatccgatcggactcgacaaaaataaattccgcaaagtggaggaagaaaacgaggaaaattcgaagatcgtggaaaat
ggccaaatgacgtacaatgacgatctcgaaatgttcacgaccattcaactgaaccaggagattgatatccgaaaaaacccgtttccactgcatatgattaac
caaaagaacgacatccctctctgtgcgcaagagttctctaagcttaccgacccactctacatcaaaaagatttgcttggagaaggtccacgactggtcgcg
atgcaacgaggacgacgtttgcgtcaaccagatcctctcgggcctgactaaccagctgttcgaggtcagcatcaaggaggacactgctattgagtaccgt
attacgcggcgccacgtcctttttcgtatctacggcaaggatgtggacgcgttgtacaatccgcttagcgaattcgaggtgtataaaactatgtcgaagtacc
gaatcgctccactgctccttaacacgtttgatggaggcagaattgaagagtggctctacggggacccgctttctatcgacgatctcaaaaacaaatctatctt
ggtgggcattgcaaatgtgcttggtaaattccacactttgtcccggaagcgacacttgcccgagcattgggacaagaccccg

<u>CRT (bp 1-456)</u>
ATGaaatttgcgtcgaagaagaataaccagaaaaacagctcgaaaaatgacgagaggtacagagagttggacaaccttgtccaggaaggtaacgga
tcgagacttggaggtggttcttgcctgggcaagtgtgcacatgtcttcaagttgatttttaaagaaatcaaagacaacatttttatttacatcctgagtatcattta
cttgtctgtctgtgtgatgaataagatttttgcaaagcgcactctcaacaagatcggaaattactctttcgtgacttccgaaacgcacaacttcatttgtatgatt
atgtttttcattgtgtactctctgttcggtaataaaaaaggaaactccaaggaacgtcaccgatctttcaaccttcagttctttgccatctccatgcttgacgcgt
gcagcgtgattctcgcatttatcgggctcacgaggacaact

<u>GSK3 (bp 1-801)</u>
ATGaagaattggcccatcgacgaggatatcaatatctacgaagaaaagaaccacacaaacaacaagaactacgtcaataacttcgagatgtcggacc
aaaaagacgaggaggaatactcgcactcgagtaataggtccgaagacgaagacgaagagcgcacgattgacaacgaaattaaccgttctcctaataaa
tcttacaagttgggaaacattatcggcaatgggtctttcggcgtcgtctacgaggccatctgcattgatactagcgaacaggttgcgatcaagaaggtcctc
caggacccacaatacaagaacagagagctcatgatcatgaagaacttgaatcatattaacatcatctatctgaaggactactactacaccgagtcctttaag
aaaaacgaaaagaatatctttctcaatgtcgtcatggagtacatcccccagaccgttcacaagtacatgaaatactacagtcgcaataatcaggctctccct
atgttcttggtgaaactctacagttaccaactttgtcgagcactctcctacattcactctaaatttatttgccaccgagaccttaaacctcagaatctgctcattga
tccgcgaacccatacgttgaagctttgcgattttggctccgctaagaaccttctcgcgggtcaacgatccgtttcttacatctgctcgcgcttctatcgcgccc
cagagctgatgcttggctctacgaactataccacacacatcgacctgtggtctctggggtgcattattgccgagatgattcttgggta

<u>HT (bp 1-618)</u>
ATGactaaaagctccaaggacatttgcagcgagaatgagggcaaaaaaaacggtaaatctggctttttctctacctctttcaagtacgttctttcggcgtg
cattgcttcgttcatttttgggtaccaggttagtgtgctcaacactatcaagaattttatcgtcgtcgagttcgaatggtgtaaaggcgagaaggatagacttaa
ttgttctaacaatacgattcagagctcgttcttgcttgcatccgtctttatcggggccgtgctcggctgcggattctctggatacctggttcagtttggtcgtcgt
ctttctctgcttattatctacaacttcttcttcctggtgtccatccttacgtcgatcacacatcactttcatactattctgtttgcaagattgctgagtggatttggcat
cggactggtgacggtgtccgtgccaatgtatatttctgagatgacccacaaggacaagaaaggggcgtacggggtgatgcaccaactcttcatcacgttc
ggcatcttcgttgcagtgatgctgggtctcgccatgggagagggtccaaaggcggacagcacagaaccgcttaccagctttgctaagctctggtgg

<u>TrxR (bp 1-609)</u>
ATGaataatgttatcagctttatcggtaactctagcaacaagtacttccagatcaatcagctccatttcatccggatcatcaataaaaacatccactcgaag
aataaccttatcaactctaacagctcgtataacgtgttctacaataaatatttcatcaagaatactttccagaataagaataagctgtccagcatctactctaagc
tcaacttctctatcaagaatatgtgtaaggataaaaacgagaaaaagaattacgagcacgtcaacgccaacgagaaaaacggctacttggccagcgaga
aaaatgagctcacgaagaacaaggttgaggagcacacttacgactacgactacgtcgttattgggggggacctgggggaatggccagtgcgaagga
ggccgccgcccacggcgcgagagtgctcctctttgactacgtgaagcccagttcgcaaggaaccaagtggggcattgggggcacatgcgtgaacgtg
ggatgcgtcccgaagaaactcatgcactacgctggacacatggggagcattttcaagctggactcgaaagcttacggatggaagttcgacaatctcaag
cacgactgg

Supplementary Table 3. List of oligonucleotides used to construct pSN150-based knockout donor vectors and validate locus-specific integration in edited *P. falciparum* lines.

Description	Nucleotide sequence
CK_RHR_F	CTAAATATATATCCAATGGCCCCTTTCCGGGCGCGCCGATCATTTATGTGTTTTCCTGGTAGCAC
CK_RHR_R	CTTCTTCGCTGATGAGTTTCTGTTGCGCCCGGGTAACATATTGCATAATTTTTTAATTTATTTCAAATTATGGGG
CK_LHR_F	TTTCAAACTTCATTGACTGTGCCGGCCGGCCGTATGTATCACATAAGTTTTGATTTTATC
CK_LHR_R	TCTTGAAAACAAGAATCTTTTTATTGTCCTGTTCTATTAGGTGCTAGATACTAATTTTAC
CK_sgRNA target site	ATGGTGGGCGTATCGAGGAA
CK_LHR integration primer_F	CACATTATTGTAAACCTGTATACGCAC
CK_RHR integration primer_R	AGAGGTATATAATTTATATATTATTATATTTACATATTATTGATGACAC
GSK3_RHR_F1	ACTAAATATATATCCAATGGCCCCTTTCCGGGCGCGCCGATATATATATATATATATATATATCAGGAAATAATTAAACTGTAAACATGG
GSK3_RHR_R1	AGATCTTCTTCGCTGATGAGTTTCTGTTGCGCCCGGGATAATGATATAAAGAATCATATGATTAAATTTTTAAATACAGC
GSK3_LHR_F2	TTCAAACTTCATTGACTGTGCCGGCCGGCCAACCCAAATAGATATACATATAAATAAATATAAATATATAATATATATTATATTTCC
GSK3_LHR_R2	GTTCTTGAAAACAAGAATCTTTTTATTGTCGTATAATTACAAAATTGACAAAAGGTAATAATCAAAAATATTAATTG
GSK3_sgRNA target site	GAGTGATCAGAAGGACGAAG
GSK3_LHR integration primer_F	TATACCATCATATTTACAATATCGTGAGTAT
GSK3_RHR integration primer_R	GTATAAAATTTATAAGCGTTTCTACTGATCCAC
TrxR_RHR_F	TATATATCCAATGGCCCCTTTCCGGGCGCGCCATGCATTTATAGTTCACTGCATTTCTCC
TrxR_RHR_R	TCACAGATCTTCTTCGCTGATGAGTTTCTGTTGCGCCCGGGATGTTTAGCCAATTCTATTGAAGCATCAC
TrxR_LHR_F	ATTGGTTTTCAAACTTCATTGACTGTGCCGGCCGGCCGTGGGTGGACTTTAAGTTTAGTT
TrxR_LHR_R	GTTCTTGAAAACAAGAATCTTTTTATTGTCGCCAAATGTGTAAAAATAAAAAAAGAGAGA
TrxR_sgRNA target site	AGGAGGAGGTCCAGGTGGAA
TrxR_LHR integration primer_F	CCGGTTTCATTAGTGTCTATATGG
TrxR_RHR integration primer_R	CACGAATAAATGATGAAATATGTGGATATGC
pSN150_LHR integration_R	CTTCGCATCTGGGCAGATGATGTC
pSN150_RHR integration_L	AGCTACCCATACGATGTTCCAG

Legend: LHR, left homology region; RHR, right homology region; F, forward; and R, Reverse.

Supplementary Table 4A. List of oligonucleotides used to construct pSN054-based knockdown donor vectors and validate locus-specific integration in edited *P. falciparum* lines.

Description	Nucleotide sequence
AAT_RHR_F	AGTGGTGTACGGTACAAACCCGGAATTCGAGCTCGGTAATTAAATTAATATGTTTTAATTAAAAAAAAAAAAAAAAAAATAAATAAATATAAATA
AAT_RHR_R	GATAAGACGAGAGATTGGGTATTAGACCTAGGGATAACAGGGTAATCATATCTTAGATCCGCTTGAC
AAT_LHR_F	TTATTGGTTTTCAAACTTCATTGACTGTGCCGGCCGGTGGGTATTAAACATAATGGTCAG
AAT_LHR_R	GCTATAAAAAATACCATCCGCAAA
AAT_sgRNA target site	GTCCACGAAATGGTCATCAT
AAT_LHR integration primer_F	TATCAAATTCGGATTCTAAGCATATTAT
AAT_RHR integration primer_R	ACAAGTGTGTATATCGTGTGGTATAA
FC_RHR_F	CGGTCTCAGTGGTGTACGGTACAAACCCGGAATTCGAGCTCGGATCCATAAGCGAAAAAAAATGATTA
FC_RHR_R	GGATAAGACGAGAGATTGGGTATTAGACCTAGGGATAACAGGGTAATAAATAAATAAATAAAGGAAGGAAGGC
FC_sgRNA target site	ATAATAAGGCTAGCTAAACA
FC_LHR integration primer_F	GTACACATTTGTATTACCTTTCAG
FC_RHR integration primer_R	GCTTTATGTTTTTCTTTTTTTGTTGTTAT
HT_RHR_F	TCTCAGTGGTGTACGGTACAAACCCGGAATTCGAGCTCGGGACCAAGTCGGTTGTATGAT
HT_RHR_R	TGGATAAGACGAGAGATTGGGTATTAGACCTAGGGATAACAGGGTAATGTGGAGGTAGTAGCATAATAAA
HT_LHR_F	ATTGGTTTTCAAACTTCATTGACTGTGCCGGCCGGCCTCTGGTTTACAACAATTTACAGG
HT_LHR_R	GTCTGATGGGAAGACAAC
HT_sgRNA target site	AAGTCCATACATAACTATGG
HT_LHR integration primer_F	TTAGTTACCGTAAGTGTTCCTATGTATA
HT_RHR integration primer_R	ACCTACTTTTGACCATATCATAATTTCTAC
pSN054_LHR integration_R	GCCCTTTATCATCATCATCTTT
pSN054_RHR integration_F	TCTTGAGGGGTTTTTTGCG

Legend: AAT, putative amino acid transporter (PF3D7_0209600); FC, ferrochelatase (PF3D7_1364900); HT, hexose transporter (PF3D7_0204700); LHR, left homology region; RHR, right homology region; F, forward; and R, Reverse. The FC LHR and recodonized regions were synthesized as a single fragment on the BioXp 3200 System (SGI-DNA).

Supplementary Table 4B. Recoded regions (*T. gondii* codon composition) used to construct pSN054-based knockdown donor vectors.

AAT recoded region (bp 4076-4506) intron 7 removed, stop codon removed
AACGAGATCAAGGAGAAGGTCAAGATCATACCCAAAGGCTGGGTCTTTTTCAGCATCTTCTTCTGGGTCTTCATCATGTTGTTGCTCTTCTTGTTTCCCTTCTTCACC
CAAACGTTCGAGAAATACTGCGCGTACGACGATCACTTTGTCGATTTCAGCATCTTGCCGAGTAAACCGCTCAAGGAGGTCCAGAACTTCAACATATTGAGCTACT
TCTACGAGTTCAAGAACATACGCAAACGCCGCAAGAAAAAAACGAAGAAGATACGCGTGGAC

FC (bp 1341-1512) stop codon removed
CTCAACTTGAACAAGGACGACTACATCCTCGTGTTCCAGAGCAAGATAAAGGGCCAGCAGTGGGTCAAGCCCTGCATAGAGGACACGATCATCCGCTTGGCGAA
GCAGGGCTACAAGCAGATCGACATAGTGTCGCCCAGCTTTAGCAGCGACTGCCTCGAGACCCTCGAGGAGATCAAGATCCACTACCAGCAGCTCTTCAGGAAGT
ACAGCAACGGCAACCTCCGCTACATCAACTGCCTCAACGACACCACCATAGGGATAAAGTTGATCATGAACTTGATCGAGCAGAACATCATCGGCTGGGTC

HT (bp 1342-1651) stop codon removed
ATAATAATAAAAAAGAGTCCAAGCATATTGTTTATCGTGTTCAGCGTGATGAGCATACTTACGTTTTTTTTCATATTCTTTTTCATAAAGGAGACGAAGGGTGGGG
AGATCGGTACCTCACCTTATATCACGATGGAAGAAAGGCAGAAACACATGACGAAAAGCGTGGTT
